# Neutrophil: A Cell with Many Roles in Inflammation or Several Cell Types?

**DOI:** 10.3389/fphys.2018.00113

**Published:** 2018-02-20

**Authors:** Carlos Rosales

**Affiliations:** Departamento de Inmunología, Instituto de Investigaciones Biomédicas, Universidad Nacional Autónoma de México, Ciudad de México, Mexico

**Keywords:** neutrophil, bacteria, infection, inflammation, cancer

## Abstract

Neutrophils are the most abundant leukocytes in the circulation, and have been regarded as first line of defense in the innate arm of the immune system. They capture and destroy invading microorganisms, through phagocytosis and intracellular degradation, release of granules, and formation of neutrophil extracellular traps after detecting pathogens. Neutrophils also participate as mediators of inflammation. The classical view for these leukocytes is that neutrophils constitute a homogenous population of terminally differentiated cells with a unique function. However, evidence accumulated in recent years, has revealed that neutrophils present a large phenotypic heterogeneity and functional versatility, which place neutrophils as important modulators of both inflammation and immune responses. Indeed, the roles played by neutrophils in homeostatic conditions as well as in pathological inflammation and immune processes are the focus of a renovated interest in neutrophil biology. In this review, I present the concept of neutrophil phenotypic and functional heterogeneity and describe several neutrophil subpopulations reported to date. I also discuss the role these subpopulations seem to play in homeostasis and disease.

## Introduction

**Figure 1:**
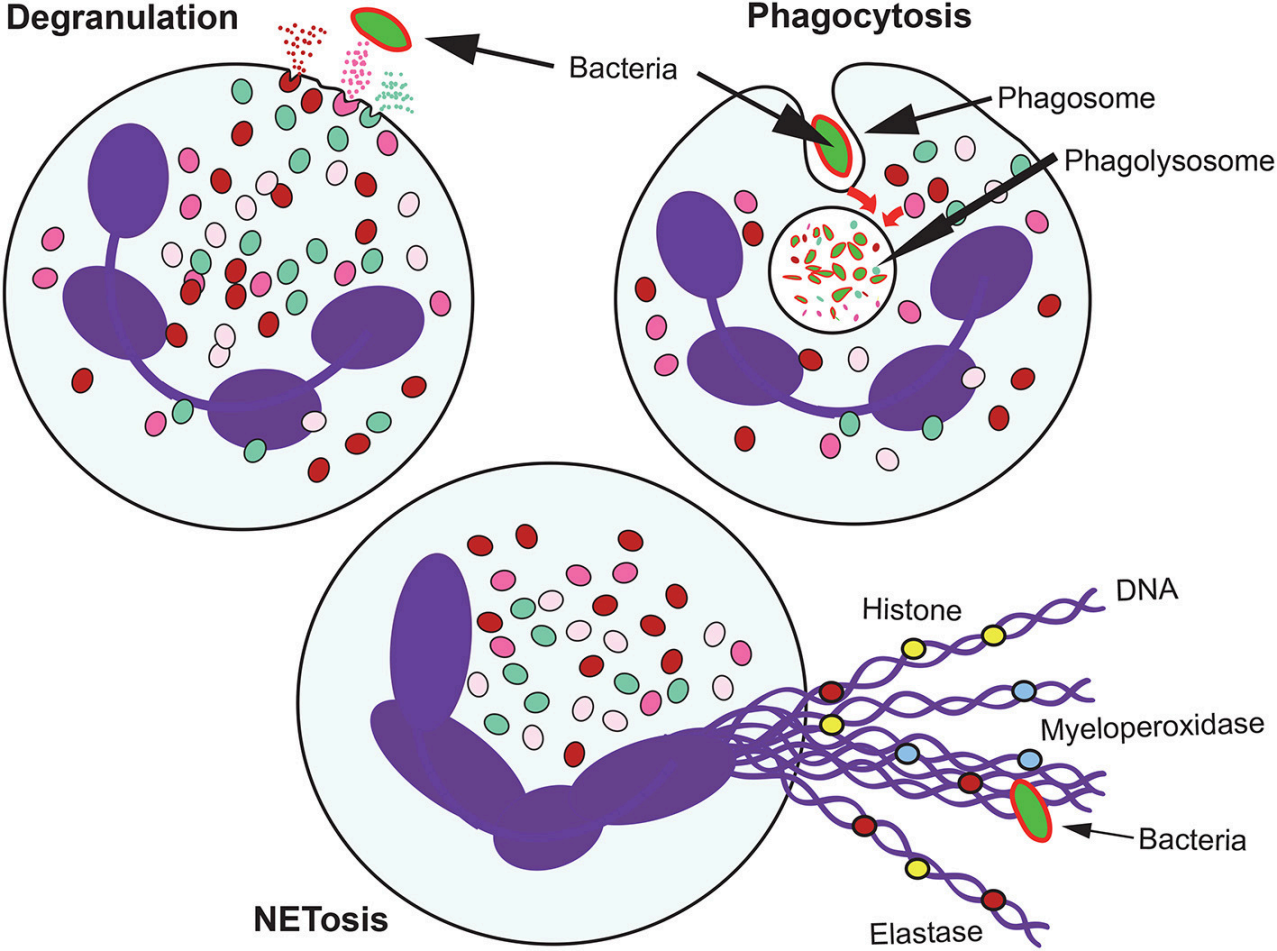
Antimicrobial mechanisms of neutrophils. When neutrophils recognize microbial pathogens, they deploy different functions to destroy them. Phagocytosis involves the ingestion of the microorganism into a phagocytic vacuole that upon maturation becomes a phagolysosome. In this new organelle, the microorganism is destroyed by the action of low pH, and degrading enzymes. Neutrophils also degranulate and release to their environment the contents of their granules. When the microorganism is too large to be ingested, neutrophil can also produce extracellular traps (NETs) formed by DNA fibers and proteins from the granules.

Neutrophils, also known as polymorphonuclear (PMN) leukocytes, are the most abundant cell type in human blood. They are produced in the bone marrow in large numbers, ~10^11^ cell per day. Under homeostatic conditions, neutrophils enter the circulation, migrate to tissues, where they complete their functions, and finally are eliminated by macrophages, all in the lapse of a day. Neutrophils are important effector cells in the innate arm of the immune system (Mayadas et al., 2014). They constantly patrol the organism for signs of microbial infections, and when found, these cells quickly respond to trap and kill the invading pathogens. Three main antimicrobial functions are recognized for neutrophils: phagocytosis, degranulation, and the release of nuclear material in the form of neutrophil extracellular traps (NETs) (Figure 1). These functions were considered, until recently, the only purpose of neutrophils. However, current research by investigators in several fields of neutrophil cell biology has revealed that neutrophils possess a much diverse repertoire of functional responses that go beyond the simple killing of microorganisms. Neutrophils respond to multiple signals and respond by producing several cytokines and other inflammatory factors that influence and regulate inflammation and also the immune system (Nauseef and Borregaard, 2014; Scapini and Cassatella, 2014). Nowadays it is recognized that neutrophils are transcriptionally active complex cells (Ericson et al., 2014) that produce cytokines (Tecchio and Cassatella, 2016), modulate the activities of neighboring cells and contribute to the resolution of inflammation (Greenlee-Wacker, 2016), regulate macrophages for long-term immune responses (Chen et al., 2014), actively participate in several diseases including cancer (Uribe-Querol and Rosales, 2015; Mishalian et al., 2017), and even have a role in innate immune memory (Netea et al., 2016).

The multitude of neutrophil functional responses is induced by transcriptional activation and by changes in expression of surface molecules or activity. These phenotypic changes are usually detected in only a subset of neutrophils, suggesting that great neutrophil heterogeneity exists (Beyrau et al., 2012). Neutrophils display different phenotypes from the time they leave the bone marrow and enter the circulation (fresh neutrophils) to the time they disappear from the circulation (aged neutrophils). This shift in phenotype is known as aging, since it takes place within a single day, and results in various neutrophils with distinct properties (Adrover et al., 2016). In addition, the microenvironment in different tissues can induce neutrophils to acquire specialized functions. Thus, the fact that neutrophils can display many functional phenotypes further supports the existence of several neutrophil subsets.

Cancer is a particular condition, in which the number of neutrophils in circulation increases, and the phenotype of these cells changes along tumor progression. In advanced cancer, several subpopulations of circulating neutrophils with different characteristics of maturity, tumor cytotoxicity, and immune suppression have been described (Sagiv et al., 2015), including the granulocytic myeloid derived suppressor cells (G-MDSC). However, these different cell types are not clearly defined and their existence as bona fide neutrophil subsets or even a complete different cell type is today a controversial topic. For most researchers, it is clear that the differences in plasma membrane proteins and functional responses of neutrophils under diverse settings are strong evidence for the remarkable plasticity of neutrophils. Yet, this neutrophil heterogeneity is not supported by consensus criteria by which to define populations of neutrophils in blood and in tissues. Additionally, the stability of observed phenotypes in these subpopulations is not clear. Are these bona fide neutrophil subsets or just various activating stages displayed in response to local factors?

It is the purpose of this review to highlight the differences in functional responses of neutrophils and other cell types, such as G-MDSC, that may or may not be subpopulations of circulating neutrophils. We present the characteristics of the different neutrophil types, describe their functions, and discuss the possible relations among them.

## Neutrophil life cycle

Neutrophils represent about 70% of all leukocytes and more than 10^11^ cells are produced every day in the bone marrow (Dancey et al., 1976). From there, neutrophils enter the blood where they circulate until they leave into tissues. Once neutrophils reach the end of their lifespan within tissues, they are cleared mostly by macrophages through the process of phagocytosis (Bratton and Henson, 2011). Despite this impressive turnover, the number of neutrophils in circulation remains relatively constant thanks to a fine balance between production and elimination (neutrophil homeostasis; von Vietinghoff and Ley, 2008). In addition, neutrophils actively change to be able to perform special functions at different times or places.

### Granulopoiesis

**Figure 2:**
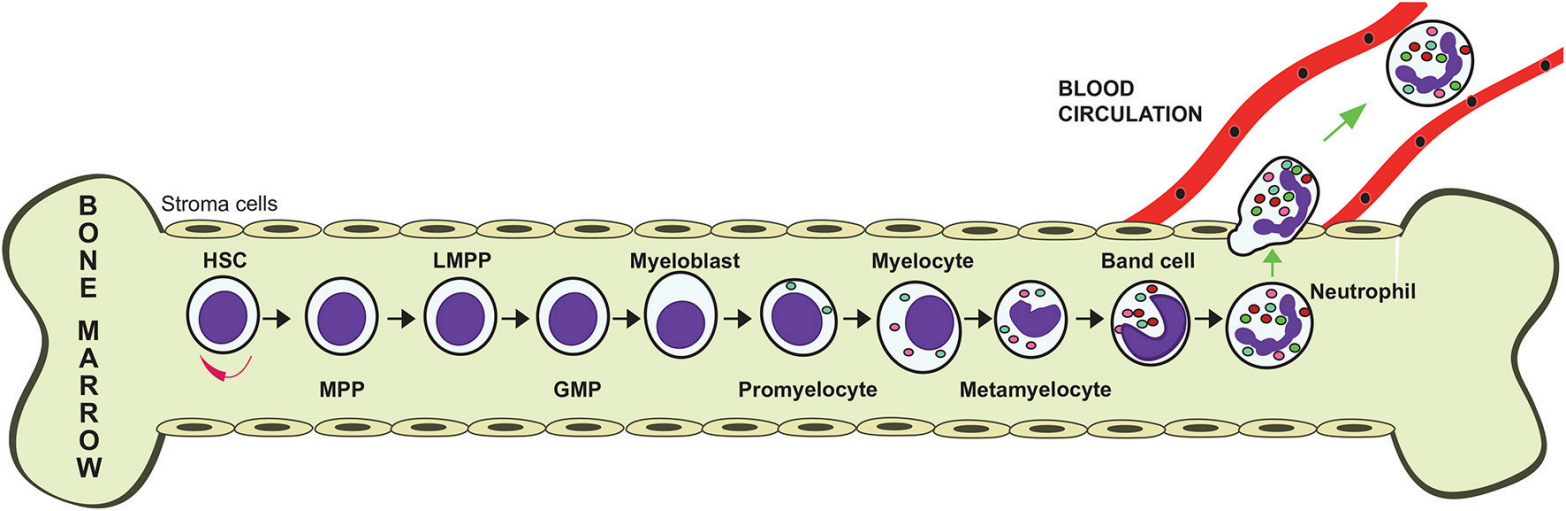
Granulopoiesis. Neutrophils are produced in the bone marrow. From a self-renewing hematopoietic stem cell (HSC), a multipotent progenitor (MPP) cell is formed. MPPs give rise to lymphoid-primed multipotent progenitors (LPMP), which differentiate into granulocyte-monocyte progenitors (GMP). These GMPs, under control of the granulocyte colony-stimulating factor (G-CSF) commit to neutrophil generation by turning into myeloblasts, which then follow a maturation process that includes the stages of promyelocyte, myelocyte, metamyelocyte, band cell, and finally a mature neutrophil.

Neutrophils are produced in large numbers in the bone marrow from hematopoietic stem cells (Görgens et al., 2013). These cells differentiate into multipotent progenitor (MPP) cells that cannot self-renew themselves. MPPs then transform into lymphoid-primed multipotent progenitors (LMPPs), which differentiate into granulocyte–monocyte progenitors (GMPs). These GMPs, under control of the granulocyte colony-stimulating factor (G-CSF) commit to neutrophil generation by turning into myeloblasts (Figure 2). These cells then follow a maturation process that includes the stages of promyelocyte, myelocyte, metamyelocyte, band cell, and finally a mature neutrophil (von Vietinghoff and Ley, 2008) (Figure 2). During differentiation, the developing neutrophil changes its nucleus from a round shape into a banded and then a lobulated morphology, and also the expression of various receptors. The integrin α4β1 (VLA4) and the CXC chemokine receptor 4 (CXCR4) are downregulated, while CXCR2 and Toll-like receptor 4 (TLR4) are upregulated. The bone marrow stroma cells express vascular cell adhesion molecule 1 (VCAM1), a ligand for VLA4, and the chemokine stromal-derived factor-1/SDF-1 (CXCL12), a ligand for CXCR4, in order to retain the progenitor cells in the bone marrow. Mature neutrophils also contain granules and secretory vesicles that store specific proteins relevant to their functions (Häger et al., 2010). These granules are formed at particular differentiation stages. Primary (azurophil) granules are found at the myeloblast to promyelocyte stage. Secondary (specific) granules are detected at myelocyte and metamyelocyte stages. Tertiary (gelatinase) granules are found at the band cell stage. Finally, secretory vesicles are detected only in mature neutrophils (Figure 2). These granules store an arsenal of antimicrobial enzymes, including elastase, myeloperoxidase, cathelicidins, defensins, and matrix metalloproteinases, which are used to destroy invading pathogens.

### Neutrophil exit from the bone marrow

Once neutrophils mature they can leave the bone marrow into circulation. The release of neutrophils is tightly controlled since only 1 or 2% of all neutrophils in the body are found in the blood under normal homeostatic conditions. Mature neutrophils are kept in the bone marrow through the action of two chemokine receptors, CXCR2 and CXCR4. Osteoblasts and other bone marrow stromal cells produce CXCL12 and keep CXCR4-expressing neutrophils in the bone marrow. G-CSF induces neutrophil exit from the bone marrow by interfering with the CXCR4-CXCL12 interaction (Summers et al., 2010). In addition, ligands for CXCR2, such as CXCL1, CXCL2, CXCL5, and CXCL8 (in humans) are expressed by endothelial cells outside the bone marrow when neutrophils need to be mobilized into the blood (Eash et al., 2010; Köhler et al., 2011). G-CSF prompts the release of neutrophils by inducing upregulation of CXCR2 ligands on megakaryocytes (Köhler et al., 2011), reduced expression of CXCL12 by bone marrow stroma cells (Petit et al., 2002; Semerad et al., 2005), and also reduced expression of CXCR4 on neutrophils themselves (Kim et al., 2006).

Outside the bone marrow, neutrophil production is also regulated by a cytokine network that involves interleukin (IL)-23 produced by phagocytes and IL-17 produced by T lymphocytes. In this mechanism, macrophages and also dendritic cells phagocytose apoptotic neutrophils (Gordy et al., 2011; Jiao et al., 2014) leading to a reduction on IL-23 (Stark et al., 2005), which in turn controls expression of IL-17 by T lymphocytes (Gaffen et al., 2014). Because, IL-17 promotes granulopoiesis and neutrophil release by up-regulation of G-CSF (von Vietinghoff and Ley, 2008), the lower levels of IL-17 then result in reduced expression of G-CSF, and a steady-state release of neutrophils. During inflammation, IL-1 can also stimulate neutrophil production through the IL-17-G-CSF axis (Ueda et al., 2009), and neutrophils themselves create a positive loop for neutrophil recruitment. Neutrophils can produce IL-17 (Eskan et al., 2012), and attract IL-17-producing T lymphocytes (Th17 cells) (Weaver et al., 2013). In turn, Th17 cells recruit more neutrophils (Pelletier et al., 2010; Zenobia and Hajishengallis, 2015). Recently, it was also found that microbiota can induce neutrophil production by increasing IL-17 production (Deshmukh et al., 2014).

### Neutrophil trafficking and clearance

Neutrophils from the blood can be mobilized to sites of infection or inflammation through the process known as the leukocyte adhesion cascade (Ley et al., 2007; Chavakis et al., 2009). Endothelial cells of blood vessels close to the affected site get activated and express adhesion receptors such as E-, and P-selectins. These receptors bind glycoprotein ligands on neutrophils, causing them to roll on the endothelium. Next, the neutrophil is activated by chemokines, which induce a high affinity state in β2 integrins. Binding of integrins to their ligands such as intercellular adhesion molecule-1 (ICAM-1) and ICAM-2 on endothelial cells causes firm adhesion of the neutrophil. Next the neutrophil transmigrates into peripheral tissues (Hajishengallis and Chavakis, 2013). Once neutrophils are in the peripheral tissues, they follow gradients of chemoattractans such as formyl-methionyl-leucyl-phenylalanine (fMLF), and the anaphylatoxin C5a to complete their functions (Kolaczkowska and Kubes, 2013).

The constant numbers of neutrophils in the circulation are also controlled by central signals delivered by the sympathetic nervous system. In this case, adrenergic nerves induce the temporal expression of adhesion molecules on endothelial cells, allowing neutrophils to bind the endothelium and leave the circulation (Scheiermann et al., 2012). This regulation of neutrophil migration into tissues follows a circadian pattern (Scheiermann et al., 2013; see below).

Once in tissues, neutrophils undergo apoptosis and are finally cleared through phagocytosis by resident macrophages and dendritic cells. Senescent neutrophils in blood upregulate expression of CXCR4, which allows them to return to the bone marrow for final clearance (Martin et al., 2003). The clearance of apoptotic neutrophils is also important for controlling neutrophil production in the bone marrow (Stark et al., 2005). Phagocytosis of apoptotic neutrophil triggers an anti-inflammatory response characterized by a reduction in IL-23 by macrophages. As described above, less IL-23 leads to reduced IL-17 levels and to less G-CSF production, and finally, in consequence to reduced granulopoiesis (Stark et al., 2005).

## Neutrophil subpopulations in health

The previous description of the neutrophil life cycle gives the idea that these cells are produced in the bone marrow, go to the circulation, migrate to sites of infection or inflammation, execute their antimicrobial functions, and then quietly are cleared by tissue-resident macrophages. This simple view for neutrophils being only pathogen-killing cells, is far from the complex behavior neutrophils actually display. It was already mentioned that in fact neutrophils are transcriptionally active cells with the potential to change the expression of several membrane molecules, and to produce cytokines (Tecchio et al., 2014; Tecchio and Cassatella, 2016), and consequently neutrophils are capable of performing different cell functions depending on the tissues where they are found (Borregaard, 2010; Mayadas et al., 2014; Nauseef and Borregaard, 2014). Thus, neutrophils do not seem to be a homogeneous population that always behaves the same. In fact, the existence of several subpopulations of neutrophils has been suggested in various conditions of health and disease.

### Neutrophil subpopulations in the circulation

In normal conditions, neutrophils remain in circulation for just few hours (the half-life is estimated at 6–12 h) before they leave into tissues (Summers et al., 2010). During this time, neutrophils appear to change their phenotype. Looking at neutrophils in circulation of healthy mice every 4 h over the course of a day, it was found that these cells change their morphology and phenotype (Casanova-Acebes et al., 2013). Freshly released neutrophils from the bone marrow undergo several changes that accumulate until the cells begin to move from the circulation into the tissues. These changes in neutrophil phenotype from the time they are released from the bone marrow (fresh neutrophils) until they leave the circulation (aged neutrophils) in the absence of inflammation are referred to as aging (Adrover et al., 2016). The number of total neutrophils oscillates in a circadian way. Fresh neutrophils are released into the blood when the mice begin their activity phase, and aged neutrophils are cleared at the end of their resting stage (Casanova-Acebes et al., 2013).

Previous *in vitro* studies on neutrophil aging indicated that there is a spontaneous upregulation of the receptor CXCR4 in cells that are kept in culture. Freshly isolated blood neutrophils present this increase in CXCR4 expression after only 4 h in culture (Nagase et al., 2002). As mentioned before, the chemokine CXCL12 produced by bone marrow stroma cells functions as a retention signal for neutrophils. Thus, re-expression of CXCR4 on neutrophils is thought to encourage “senescent” neutrophils to return to the bone marrow (Martin et al., 2003). However, in mice with CXCR4-deficient myeloid cells, no changes in neutrophil clearance were observed (Eash et al., 2009), suggesting that other organs besides the bone marrow also contribute to neutrophil clearance (Adrover et al., 2016). In addition, neutrophils in culture also downregulate the expression of CXCR2, which has opposite effects to CXCR4 and promotes the release of cells from the bone marrow (Eash et al., 2010). Therefore, it seems that these phenotypic changes prepare neutrophils to leave the circulation into tissues.

**Figure 3:**
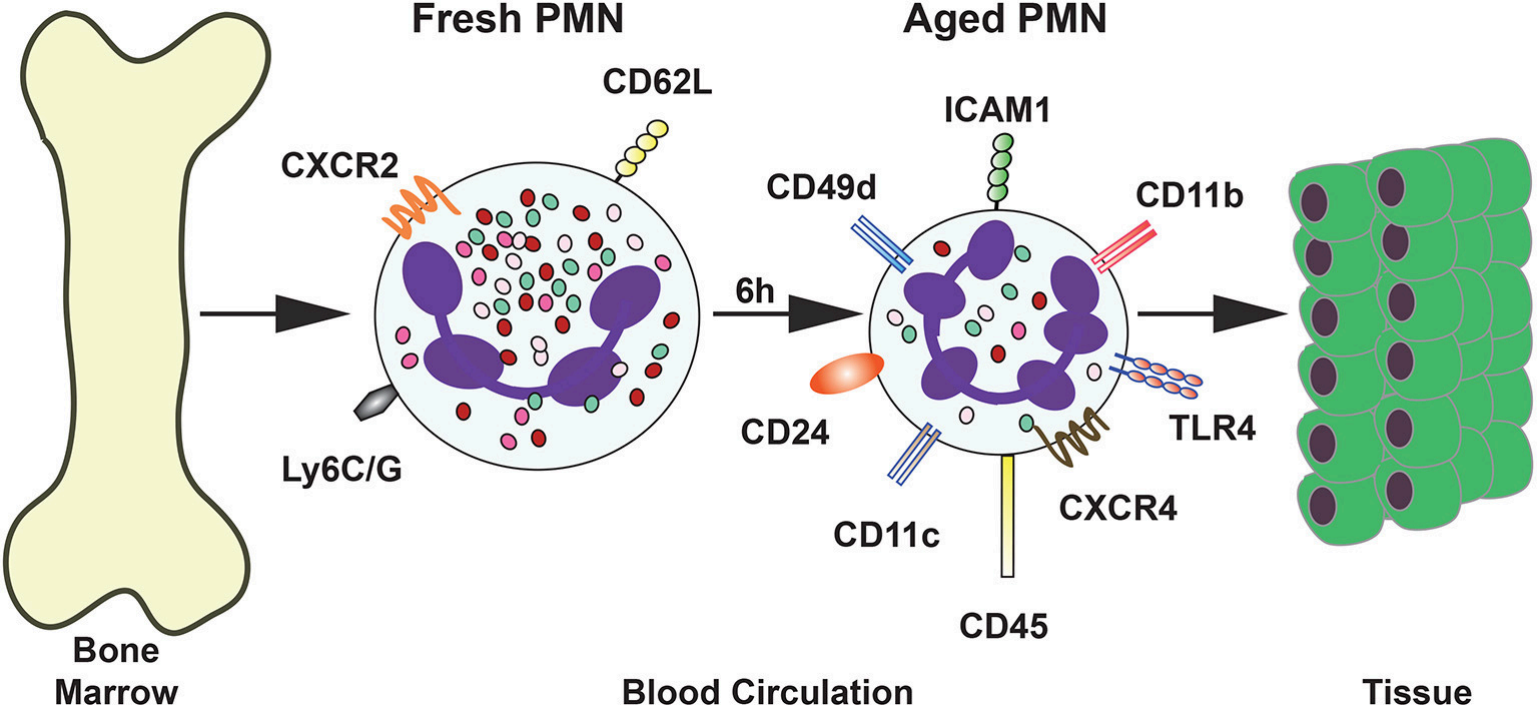
Phenotype of aged neutrophils. Upon release from the bone marrow, a “fresh” neutrophil (PMN) express the surface molecules CD62L and CXCR4. After several (4–6 h) hours in the circulation neutrophils change the expression of many surface molecules. The new phenotype is described as “aged” neutrophils. These aged neutrophils are then cleared from the blood by migration into tissues or by returning to the bone marrow.

*In vivo* studies have identified other phenotypic changes in neutrophils. In mice, neutrophils that were forced experimentally to stay longer in the circulation presented lower expression of L-selectin (CD62L), together with a higher expression of CXCR4 (Casanova-Acebes et al., 2013). These aged neutrophils appeared in the circulation following circadian oscillations over time. They increased in numbers during the day (when mice are at rest), and disappeared in the evening, when mice begin their active phase (Casanova-Acebes et al., 2013). Other molecules are also expressed al higher levels on the cell membrane of these aged neutrophils, including CD11b (α_M_) and CD49d (α4), the alpha subunits for integrins Mac-1 and VLA4, respectively. These integrins are involved in adhesion to activated endothelial cells at sites of inflammation. In contrast, the expression of the molecule CD47, a “don't eat me” signal for apoptotic cells (Jaiswal et al., 2009), was reduced on the membrane of these neutrophils. This would suggest that aged neutrophils are more easily phagocytosed by macrophages. However, in a more recent study, the expression of CD47 was not reduced (Zhang et al., 2015). More recently, the surface expression of other molecules has also been found increased in aged neutrophils. Some of these molecules include TLR4, ICAM-1, CD11c, CD24, and CD45 (Zhang et al., 2015) (Figure 3). Opposite to this, the mouse neutrophil marker Ly6G was also reduced in aged cells (Zhang et al., 2015) (Figure 3). Together with the changes in surface expression of these molecules, the cells presented morphological changes. Aged neutrophils are smaller, contain fewer granules, and display a granular multilobullar nucleus (Casanova-Acebes et al., 2013; Figure 3).

In addition, transcriptomic analysis of aging neutrophils revealed that several signaling pathways are different from those active pathways in fresh neutrophils. Signaling related to cell activation, microbial detection, adhesion, migration, and cell death is altered (Zhang et al., 2015). These changes in aged neutrophils are similar to changes in neutrophils at inflammation sites. Thus, it might be that aged neutrophils are in an activated state (Adrover et al., 2016). Together these reports suggest that neutrophils spontaneously change their phenotype in circulation, such that different surface molecules may facilitate migration into tissues. At present, it is not known how these differences in receptor expression may control neutrophil clearance into particular tissues, but a similar mechanism to the one controlling neutrophil exit from and return to the bone marrow is likely to be at work. Identifying particular neutrophil phenotypes will certainly help us understand how these cells are directed to various parts of the body.

### Neutrophil subpopulations in tissues

In normal homeostasis, neutrophils are found in many tissues (von Vietinghoff and Ley, 2008, 2009), where they perform specialized functions. For the most part, there is very little knowledge on how neutrophils are directed to the different organs, and the particular functions they perform in each tissue. Evidence suggests that indeed neutrophils display different phenotypes in various organs. For example, in the lung a large number of neutrophils accumulate adhered to the vascular lumen and in the interstitial space. These neutrophils are kept in the tissue by a CXCR4-dependent mechanism (Devi et al., 2013). Because aged neutrophils express more CXCR4 it is possible that they preferentially migrate to the lungs (Adrover et al., 2016). Similarly, in the spleen many neutrophils are found in the marginal zone, producing cytokines that induce somatic hypermutation and antibody production by marginal B lymphocytes (Puga et al., 2011). Also, neutrophils in spleen display the phenotype CD62L^low^ CD11b^hi^ ICAM-1^hi^, and have a tendency to produce NETs (Cerutti et al., 2013), similar to aged neutrophils in the circulation (Summers et al., 2010; Casanova-Acebes et al., 2013).

Another subpopulation of neutrophils has been found to preferentially move into lymph nodes to interact with T lymphocytes, carrying antigens to the lymph nodes and mediate T cell activation (Duffy et al., 2012; Hampton and Chtanova, 2016). These neutrophils can selectively migrate to the lymph node because they express the CCR7 receptor (Beauvillain et al., 2011), as well as the integrin LFA-1 and the chemokine receptor CXCR4. These receptors are also involved in neutrophil trafficking through afferent lymphatics (Gorlino et al., 2014).

Yet another subtype of neutrophils in tissues is capable of inducing angiogenesis. These neutrophils display the phenotype CD49d^hi^ CXCR4^hi^ VEGFR1. The latter is the receptor for vascular endothelial growth factor-A (VEGF-A). These neutrophils are efficiently recruited to non-vascularized tissues under hypoxia conditions, and promote angiogenesis (Massena et al., 2015). Interestingly, this proangiogenic subset of neutrophils is similar to neutrophils that support tumor vascularization (Jablonska et al., 2010; see later).

The examples described above indicate that indeed neutrophils can display great phenotypic and functional heterogeneity, and support the idea that at least some of the neutrophil subtypes may derive from aging neutrophils migrating into tissues.

### Migrating neutrophil subpopulations

Some neutrophils have been observed to perform reverse transendothelial migration (rTEM) or reverse interstitial migration (rIM), depending on their initial location (Nourshargh et al., 2016). Neutrophils doing rTEM have been observed in mice (Woodfin et al., 2011), whereas neutrophils doing rIM have been seen only in the transparent zebra fish model (Mathias et al., 2012). Neutrophils performing rTEM present the phenotype ICAM-1^hi^ CXCR1^low^, which is different from the phenotype ICAM-1^low^ CXCR1^hi^ of circulatory neutrophils and the phenotype ICAM-1^low^ CXCR1^low^ of neutrophils in tissues (Buckley et al., 2006).

### Neutrophil subpopulations induced by the microbiota

Neutrophils can modify their functional responses after being exposed to multiple factors, through the process named neutrophil priming (Downey et al., 1995; El-Benna et al., 2016). Recent studies in mice suggest that microbial products derived from the microbiota can induce neutrophil diversity. For example, diaminopimelic acid-bearing peptides, which are recognized by the nucleotide—binding oligomerization domain containing 1 (NOD1) receptor, modify the lifespan of neutrophils (Hergott et al., 2016) and prime neutrophils for improved antimicrobial function (Clarke et al., 2010). In addition, endotoxins from the gut microbiota can enter the blood circulation and influence neutrophil aging (Zhang et al., 2015) and also the development of B cell-helper neutrophils in the spleen (Puga et al., 2011). Thus, neutrophils can display different phenotypes after priming by the microbiota.

## Neutrophil subpopulations in disease

As discussed earlier, it is becoming increasingly apparent that neutrophils are much more than just microbe-killing cells. They can display several phenotypes and perform a wide array of cellular functions. Several subsets of neutrophils are found in tissues under homeostatic conditions. We still have much to learn on how these different neutrophil subtypes are generated and recruited to tissues. In addition, various subsets of neutrophils with distinct properties are also detected in pathological conditions particularly in inflammation and in cancer (Silvestre-Roig et al., 2016; Yang et al., 2017).

### Neutrophil subpopulations in inflammation

Neutrophils are the first cell type recruited to sites of inflammation. From there, they can switch phenotypes and generate various subpopulations with different cell functions. Neutrophils can also interact, directly, or via cytokines and chemokines, with other immune cells to modulate both innate and adaptive immune responses. There is not a complete understanding of these subpopulations of neutrophils, but some clear examples showing that bona fide inflammatory subsets occur are mentioned next.

Upon infection with an antibiotic-resistant *Staphylococcus aureus*, two clear subsets of murine neutrophils can be observed. They differ in cytokine production, macrophage activation potential, expression of TLR, and expression of surface molecules. These subsets were named PMN-1 and PMN-2 (Tsuda et al., 2004). PMN-1 cells produce IL-12; classically activate macrophages, express TLR2/TLR4/TLR5/TLR8, and CD49d^hi^ CD11b^low^. In contrast, PMN-2 cells produce IL-10; alternatively activate macrophages, express TLR2/TLR4/TLR7/TLR9, and CD49d^low^ CD11b^hi^ (Tsuda et al., 2004). In systemic inflammation condition, another subset of neutrophils is generated with low doses of endotoxin. These cells have a hypersegmented nucleus and display the phenotype CD62^low^ CD11b^hi^ CD11c^hi^, which is similar to the one described for murine aged neutrophils (Casanova-Acebes et al., 2013; Zhang et al., 2015). Also, they are capable of inhibiting T lymphocytes by direct cell contact involving the integrin Mac1, and by local delivery of reactive oxygen species (ROS) (Pillay et al., 2012).

In certain organs such as liver and adipose tissue, few neutrophils are detected in normal homeostatic conditions. However, upon an inflammatory state induced by experimental obesity, neutrophil numbers increase rapidly and a metabolic imbalance is slowly generated (Talukdar et al., 2012). First, neutrophils release elastase from azurophilic granules. This enzyme can destroy insulin receptor substrate 1 (IRS1) in adipocytes and hepatocytes, and in consequence induce insulin resistance and lipogenesis (Talukdar et al., 2012). Supporting a direct role for this neutrophil subtype in metabolic disorders was the observation that altered levels of elastase or its inhibitor (1-antitrypsin) are associated with metabolic syndrome and the onset of diabetes (Mansuy-Aubert et al., 2013).

### Neutrophil subpopulations induced by metabolic deregulation

As mentioned before, neutrophil effector functions are markedly enhanced after priming. When certain metabolic functions are altered, neutrophils can be primed to present stronger pro-inflammatory responses.

In hyperglycemia human and mouse neutrophils are primed to undergo NETosis (Wong et al., 2015). First, neutrophils in circulation respond to high glucose levels by releasing S100 calcium-binding proteins A8 (S100A8) and S100A9, which interact with the receptor for advanced glycation end products (RAGE), and induce macrophages and GMPs in the bone marrow to secrete G-CSF (Kraakman et al., 2017). In consequence, production of neutrophils is enhanced (Xiang et al., 2012). These new released neutrophils are primed for ROS production and formation of NETs (Wong et al., 2015). Similarly, during hypercholesterolemia, neutrophils showed a primed state characterized by elevated ROS production, increased release of myeloperoxidase (MPO) and increased expression of CD11b (Mazor et al., 2008). Together, these reports show that hyperglycemia and hyperlipidemia generate primed, proinflammatory neutrophils that may contribute to diabetes, adipose tissue inflammation, and cardiovascular inflammation.

### Neutrophil subpopulations and NET formation

NETosis, the process for producing NETs can be activated by multiple types of microorganisms (Fuchs et al., 2007; Yipp et al., 2012). Yet, the capacity of neutrophils to undergo NETosis can vary with physiological states, suggesting a neutrophil diversity that could be clinically relevant. In fact, several reports indicate that NETs can influence thrombosis (Fuchs et al., 2010) and vascular inflammation (Kessenbrock et al., 2009; Chistiakov et al., 2015), cancer (Berger-Achituv et al., 2013; Garley et al., 2016) and autoimmunity (Stephenson et al., 2016). As mentioned before some metabolic conditions associated with states of chronic inflammation, can increase neutrophil predisposition to form NETs. Hence, neutrophils from diabetic patients (Wong et al., 2015) and from systemic lupus erythematosus (SLE) patients (Garcia-Romo et al., 2011; Villanueva et al., 2011) have been shown to be more prone to NET formation.

Nowadays NETs have been described as a player of several pathophysiological processes, including vascular diseases, such as atherosclerosis and venous thrombosis (Qi et al., 2017; Bonaventura et al., 2018), and inflammatory pathologies, such as gout and pancreatitis (Hahn et al., 2016).

Atherosclerosis is a cardiovascular disease accompanied by chronic vascular wall inflammation and endothelial cell dysfunction (Gisterå and Hansson, 2017). Hyperlipidemia can damage endothelial cells, promoting lipid deposition and plaque formation. This usually characterizes the onset of atherosclerosis. Hyperlipidemia and also hypercholesterolemia induce neutrophilia, which is positively associated with atherosclerotic plaque burden (Drechsler et al., 2010). Neutrophils attach themselves to atherosclerotic plaques, primarily through NETs formation, where cholesterol crystals function as danger signals, inducing IL-1β-mediated NETs release from neutrophils. Then, components of NETs, such as cathepsin G and cathelicidin-related antimicrobial peptide (CRAMP), can attract monocytes and macrophages to plaques (Döring et al., 2012; Wang et al., 2014). NETs can also regulate cytokine production from macrophages in atherosclerosis (Warnatsch et al., 2015), and induce endothelial dysfunction directly by activation and damage of endothelial cells (Knight et al., 2014). Furthermore, proteinases from NETs contribute to plaque instability (Hansson et al., 2015). These reports indicate that NETs directly participate in rupture of atherosclerotic plaques (Döring et al., 2017), which triggers platelet aggregation and fibrin deposition at the initial site of atherothrombosis. After plaque rupture, thrombin-activated platelets interact with neutrophils at the injured site inducing more formation of NETs (Stakos et al., 2015). Thus, neutrophils and NETs are major contributors to atherothrombosis. Different from arteries, thrombosis in veins is usually initiated by endothelial injury (Di Nisio et al., 2016) triggered by alteration in the blood flow or endothelial dysfunction (Xu et al., 2017). Subsequently, damaged endothelial cells secrete massive amounts of von Willebrand factor and P-selectin, which adhere to platelets and recruit leukocytes (Etulain et al., 2015; Michels et al., 2016). Platelets then interact directly with neutrophils and promote the production of NETs (Clark et al., 2007). NETs can also stimulate the activation of coagulation cascades (Brill et al., 2012) and not only platelet adhesion (Massberg et al., 2010), but also erythrocyte adhesion (Fuchs et al., 2010). Reciprocally, NETs induce endothelial cell activation through NET-derived proteases, histones, and defensins (Saffarzadeh et al., 2012), creating a positive feedback loop for thrombosis. These findings and studies in mice showing an association between the risk of venous thrombosis and high neutrophil counts (Ramacciotti et al., 2009), confirm that NETs make a substantial contribution to maintenance of venous thrombi.

Another inflammation process in which NETs play an important role is pancreatitis. Acute pancreatitis is an inflammatory disorder of pancreas for which no specific treatment is available. Important risk factors of acute pancreatitis are formation of gallstones and alcohol abuse (Spanier et al., 2008). The most severe cases of the disease are associated with mortality, with acute respiratory distress syndrome being the most frequent cause of death in the early phase of the disease (Pandol et al., 2007). Obstruction of the pancreatic duct causes blockage of pancreatic secretion, which is accompanied by disorders in organelle function of pancreatic acinar cells (Gukovskaya et al., 2016). These disorders promote co-localization of zymogens-containing vesicles and lysosomes leading to formation of co-localization organelles. In co-localized organelles cathepsin B activates trypsinogen to trypsin (Halangk et al., 2000; Van Acker et al., 2002), which mediates acinar cell death and thus causing severe inflammation of the pancreas (Lankisch et al., 2015; Manohar et al., 2017). The destroyed tissue will eventually be replaced by fatty tissue, typical of chronic pancreatitis (Braganza et al., 2011), if the original acute pancreatitis is not resolved (Braganza et al., 2011). Neutrophils infiltrate the pancreatic parenchyma during this acute inflammatory response (Lankisch et al., 2015), and can augment trypsinogen activation via ROS. Both trypsin activation and pancreatic injury were reduced in NADPH oxidase-deficient mice(Gukovskaya et al., 2002). In addition, infiltrating neutrophils aggravate inflammation by releasing NETs in the pancreas and at the sites of systemic injury, namely, the lungs (Merza et al., 2015). NETs incubated *in vitro* with pancreatic acinar cells led to trypsin activation in these cells, degradation of the NETs by treatment with deoxyribonuclease (DNAse) abolished the trypsin activation, reduced local acinar damage, and systemic inflammation (Merza et al., 2015). Furthermore, neutrophils enter the pancreatic ducts and there they form large deposits of NETs, also known as aggregated NETs (aggNETs). AggNETs in turn obstruct secretory flow and thereby perpetuate inflammation (Leppkes et al., 2016). Therefore, NETs formed after an initial inflammatory stimulus become the activators of further inflammation in the pancreas.

Contrary to the situations mentioned above, Gout is a disease where NETs appear to have a positive effect. Gout is an acute inflammatory reaction originated from precipitation of uric acid in the form of needle-shaped monosodium urate (MSU) crystals (So and Martinon, 2017). Aggregates of MSU crystals known as tophi, induce inflammation in the joints and tissues. Local immune cells such as macrophages and dendritic cells take up the crystals via phagocytosis. The MSU-containing phagosomes then fuse with lysosomes. The low pH in phagolysosomes causes a massive release of sodium and consequently raises intracellular osmolarity, which is balanced by passive water influx through aquaporins. This process dilutes intracellular sodium and potassium concentrations. The low potassium is a trigger for activation of NLRP3 inflammasomes (Schorn et al., 2011). Inflammasome activation by MSU crystals has been considered a response to “danger,” since damaged cells release urate and ATP into the environment (Busso and So, 2012). As a consequence of inflammasome activation, proIL-1β is cleaved to release active IL-1β and other pro-inflammatory cytokines (Kingsbury et al., 2011; So and Busso, 2014). Cytokine release then leads to a rapid and dramatic recruitment of neutrophils. Recruitment of neutrophils is further mediated by CXCR2, CXCL-8, CXCL-1, CXCL-2, and CXCL-3 (Terkeltaub et al., 1998). This neutrophil influx is accompanied by the infamously intense clinical symptoms of inflammation during an acute gout attack (So and Martinon, 2017). Due to the intense local inflammation, cytokines produced in large quantities can also enter the circulation, resulting in an acute phase response that can trigger fever and leukocytosis (Maueröder et al., 2015). Continuous recruitment of neutrophils to the site of inflammation results in very high neutrophil densities (Shah et al., 2007). After the neutrophil concentration in the tissue exceeds a certain threshold, NETs begin to aggregate and build aggNETs in which the MSU crystals are embedded in a mesh of DNA and proteins from neutrophil granules. As such, these aggNETs block MSU crystals and also trap and degrade pro-inflammatory mediators by serine proteases attached to the DNA fibers (Schauer et al., 2014). MSU crystal-induced aggNET formation is augmented by release of ATP and lactoferrin from activated neutrophils. The release of ATP during NETs formation is of high importance since extracellular nucleotides initiate anti-inflammatory clearance of dead cells by mononuclear phagocytes (Elliott et al., 2009). In addition, lactoferrin on NETs abrogates further recruitment of neutrophils and thus contributes to the anti-inflammatory action of NETs in highly infiltrated tissues (Bournazou et al., 2009). Clearly, in this case NETs have a positive anti-inflammatory effect.

The inflammation processes described before show that NETs are much more than a simple anti-microbial tool and they can be produced in very different conditions of neutrophil activation. However, NETs are a double-edged sword. On the one hand, “bad” NETs are involved in stimulating inflammation, as shown in the obstruction of blood vessels and pancreatic ducts. On the other hand “good” NETs are able to contribute to the resolution of inflammation as shown in gout (Hahn et al., 2016).

In addition, neutrophils primed by microbiota-derived products can form NETs more easily than neutrophils newly released from the bone marrow (Zhang et al., 2015). In contrast, the formation of NETs can be blocked by phagocytosis of small microorganisms via the C-type lectin receptor Dectin-1, which acts as a sensor of microorganism size (Branzk et al., 2014). Dectin-1 downregulates the translocation of neutrophil elastase (NE) to the nucleus. This protease promotes NETosis by degrading histones in the nucleus (Branzk et al., 2014). Also, neutrophils that phagocytose apoptotic cells, lose their capacity to up-regulate β2 integrins and to respond to activating stimuli that induce NETs formation (Manfredi et al., 2015). This could explain in part why in conditions in which phagocytosis of apoptotic cells is compromised, such as SLE and anti-neutrophil cytoplasmic antibody (ANCA)-associated vasculitis, a state of persistent inflammation is observed. These findings imply that heterogeneity in neutrophil function (for example for NETs formation) is also regulated by physiological signals.

It is evident that infection and inflammation can regulate the appearance of neutrophil phenotypes with unique properties. Unfortunately, nowadays we can only (incompletely) describe the function of these neutrophil subtypes. It would become important to characterize these cells at the level of surface markers, functional responses, and transcriptional profiles in order to understand their role in multiple diseases.

### Neutrophil subpopulations in cancer

The changes in neutrophil phenotype during cancer are perhaps the most impressive and best studied so far. Neutrophils play important and contradictory roles in cancer development, as reflected by several recent reviews (Sionov et al., 2015; Swierczak et al., 2015; Uribe-Querol and Rosales, 2015; Coffelt et al., 2016; Mishalian et al., 2017). In tumor-bearing mice, the number of circulating neutrophils increases along with tumor progression. Similarly, in patients with advanced cancer counts of neutrophils in blood are also increased. It is not clear how tumors can induce neutrophilia, but a common mechanism seems to be the production by tumors of cytokines that influence granulopoiesis, including G-CSF (McGary et al., 1995), IL-1, and IL-6 (Lechner et al., 2010). The presence of elevated numbers of neutrophils in the circulation is associated with poor outcome in several types of cancers (Schmidt et al., 2005). In addition, the presence of neutrophils in tumors also seems to be an indicator of poor outcome (Sionov et al., 2015). For this reason, the counts of neutrophils in blood in relation to other leukocytes have been suggested as a prognostic value in cancer. Therefore, the neutrophil to lymphocyte ratio (NLR) was introduced as a simple and inexpensive biomarker for many types of cancer (Peng et al., 2015; Faria et al., 2016). In general, the blood NLR is high in patients with more advanced or aggressive cancers (Guthrie et al., 2013), and correlates with poor survival of patients with many solid tumors (Paramanathan et al., 2014; Templeton et al., 2014). Despite the simplicity for using the NLR, it has not been accepted in many clinical settings. One reason for this is that neutrophilia can be the result of elevated granulopoiesis and as a consequence, it is not always a bad sign for cancer progression. Another reason is that neutrophilia does not correlate with poor clinical outcome in all types of cancer. In gastric cancer, for example a high NLR is indicative of positive prognosis (Caruso et al., 2002). This is indicative of the great plasticity neutrophils have. They can directly kill tumor cells and control cancer (Yan et al., 2014), but they can also acquire a pro-tumor phenotype and favor cancer (Fridlender and Albelda, 2012). Therefore, the exact role of neutrophils within the tumor is a controversial matter (Sionov et al., 2015; Uribe-Querol and Rosales, 2015).

#### Myeloid-derived suppressor cells (MDSC)

**Figure 4:**
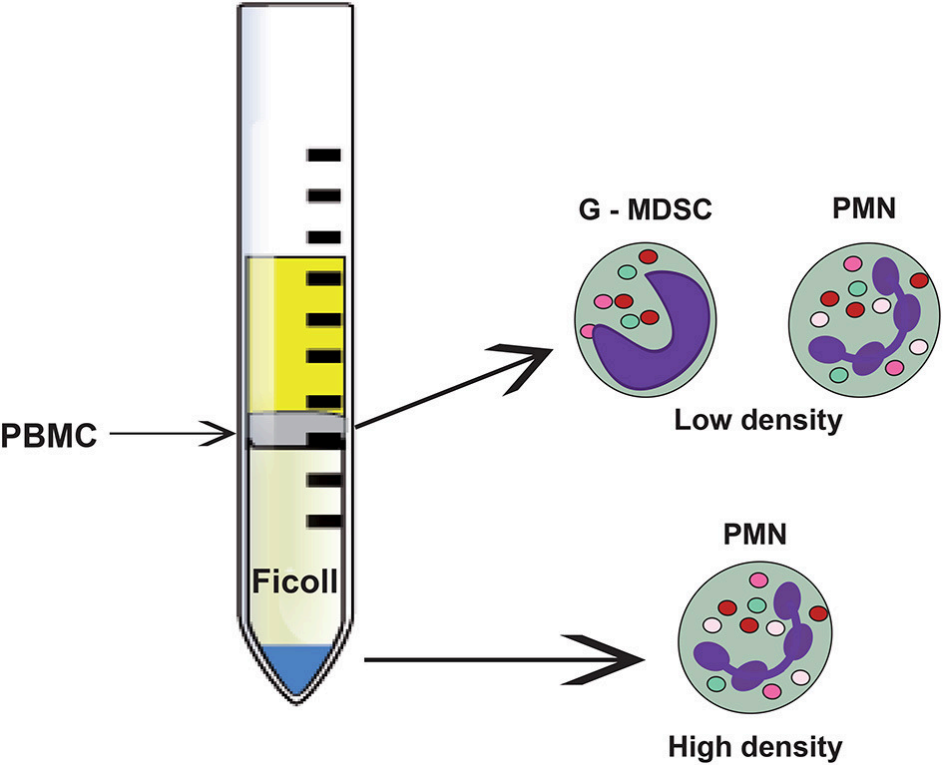
Neutrophil subsets that can be separated through density gradient centrifugation. The bulk of mature (normal) neutrophils (PMN) are denser and separate at the bottom of the density gradient. These cells, named high-density neutrophils present the classical neutrophil morphology with a lobulated nucleus and many granules. In the upper part of the gradient, the less dense peripheral blood mononuclear cells (PBMC) are separated. Among these cells, low-density neutrophils can be found. They comprise immature and mature neutrophils with immunosuppressive properties. The immature population is also called granulocytic myeloid derived suppressor cells (G-MDSC).

In several types of cancer, not only an increase in the number of neutrophils in blood is observed, but also an increase in immature myeloid cells (Brandau et al., 2013). These immature cells are at various stages of differentiation, accumulate in the spleen of tumor-bearing animals, and present an immunosuppressive phenotype that supports tumor progression (Nagaraj et al., 2010; Raber et al., 2014; Keskinov and Shurin, 2015). For this reason, they were named myeloid-derived suppressor cells (MDSC) (Peranzoni et al., 2010). These MDSC are a heterogeneous mixture of cells that can at least be divided into two subgroups: the granulocytic (G-MDSC) and the monocytic (Mo-MDSC) subgroups (Raber et al., 2014). The G-MDSC group resembles neutrophils. Hence, some researchers considerer them to be a *bona fide* phenotype of neutrophils (Pillay et al., 2013). However, the relationship among these cells is not clear since immature neutrophils do not have immunosuppressive properties (Solito et al., 2017), and neutrophils in the circulation are differentiated cells characterized by a lobulated nucleus (Pillay et al., 2013); while MDSCs are cells with clear immature morphology, including band or myelocyte-like nuclei (Pillay et al., 2013) (Figures 2, 4).

Murine neutrophils are defined as CD11b^+^ Ly6G^+^ cells (Daley et al., 2008). Murine G-MDSC are CD11b^+^ and Ly6G^+^, thus they are considered neutrophils. In contrast Mo-MDSC express CD11b and Ly6C (Brandau et al., 2013; Keskinov and Shurin, 2015), making them more monocytic- than neutrophil-like cells. In humans the problem is more complex, since the Ly6G antigen does not exist. Human mature neutrophils are defined by the phenotype CD14^−^ CD15^+^ CD16^+^ CD66b^+^ (Dumitru et al., 2012). MDSC share all these markers, making it impossible to differentiate these cells from mature neutrophils. An extended panel of six markers (adding CD11b, CD33, and HLA-DR) was used to evaluate human MDSC (Damuzzo et al., 2015). Mo-MDSC were described as CD11b^+^ CD14^+^ CD15^−^ CD33^+^ CD66b^+^ HLA-DR^−/low^; while G-MDSC were described as CD11b^+^ CD14^−^ CD15^+^ CD33^+^ CD66b^+^ HLA-DR^−^ (Brandau et al., 2013; Favaloro et al., 2014; Keskinov and Shurin, 2015). Although, these markers show differences between Mo-MDSC and G-MDSC, they do not allow for clear separation of these cells from neutrophils. At present, it is not possible to distinguish whether MDSC are indeed subpopulations of neutrophils or a separate cell type (Solito et al., 2017), but an international effort continues to find better ways for identification of these cells by flow cytometry (Mandruzzato et al., 2016).

#### Low-density neutrophils (LDNs)

An interesting subpopulation of neutrophils is the so-called low-density neutrophils (LDNs). Traditionally, neutrophils are purified by a Ficoll density gradient (Böyum, 1968; García-García et al., 2013) where they appear at the bottom of the tube (high-density fraction), separated from mononuclear cells, which are found at the interphase of plasma and Ficoll (low-density fraction) (Figure 4). In contrast, LDNs are found in the low-density fraction (Sagiv et al., 2015, 2016) (Figure 4). Interestingly, the proportion of LDNs in the low-density fraction increases with tumor growth and progression (Mishalian et al., 2017), and includes cells with mature and immature neutrophil morphology (Sagiv et al., 2015) (Figure 4). These LDNs are a subpopulation of neutrophils with characteristics and functions not well described.

Although, LDNs were first reported in the blood of patients with SLE, rheumatoid arthritis, or rheumatic fever (Hacbarth and Kajdacsy-Balla, 1986), they attracted attention only recently because they seem to be associated with cancer (Brandau et al., 2011; Sagiv et al., 2015). These LDNs have been found in many other pathological conditions including sepsis (Morisaki et al., 1992), psoriasis (Lin et al., 2011), HIV infection (Cloke et al., 2012), asthma (Fu et al., 2014), ANCA-associated vasculitis (Grayson et al., 2015), and malaria (Rocha et al., 2015). In addition, LDNs have been reported in natural pregnancy (Ssemaganda et al., 2014). During pregnancy, downregulation of T cell functions is required to ensure materno-fetal tolerance. One way to inhibit T cell function is through the enzyme arginase, which depletes _L_-arginine, an essential amino acid required for proper expression of the T cell receptor CD3 ζ chain and for T cell proliferation (Rodriguez et al., 2004; Raber et al., 2012). Arginase activity is significantly increased in the peripheral blood of pregnant women and also in term placentae (Kropf et al., 2007). The source for arginase in these tissues was identified as LDNs with the phenotype CD15^+^ CD33^+^ CD66b^+^ CD16^low^ (Ssemaganda et al., 2014). This phenotype is suggestive of a activated neutrophil (Fortunati et al., 2009), and is similar to the phenotype of G-MDSC (Favaloro et al., 2014; Keskinov and Shurin, 2015). Thus, situations of chronic inflammation and immunosuppression appear to induce neutrophil diversity. Very little is known about the function of these subpopulations of neutrophils. LDNs from SLE patients were also reported to readily form NETs (Villanueva et al., 2011), and because these NETs presented autoantigens, it has been suggested that LDNs in these patients are responsible for sustaining chronic inflammation leading to autoimmunity (Garcia-Romo et al., 2011; Khandpur et al., 2013). Similarly, it was recently reported that the number of CD66b^+^ LDNs was markedly elevated in peritoneal cavity after abdominal surgery of gastric cancer (Kanamaru et al., 2018). These LDNs readily formed NETs that selectively attached cancer cells (Kanamaru et al., 2018). These NETs could then assist the clustering and growth of free tumor cells disseminated in the abdomen.

The origin of LDNs remains unclear. Since LDNs are a mixture of cells with segmented or banded nuclei and myelocyte-like cells, one thought is that LDNs are immature neutrophils that are released from the bone marrow during chronic inflammation or immunosuppression (Denny et al., 2010; Carmona-Rivera and Kaplan, 2013). Another possibility is that these LDNs are activated neutrophils that have undergone degranulation and therefore they have a reduced density (Rocha et al., 2015; Deng et al., 2016). In mouse models of cancer, these LDNs seem to derive either from immature cells released by the bone marrow (Youn et al., 2008) or from normal-density neutrophils (Sagiv et al., 2015). It is important to notice that these LDNs are still not properly characterized. The immunosuppressive function of these cells has not been directly determined and the transition of mature (normal density) to LDNs appears to involve an increase in volume rather than degranulation (Sagiv et al., 2015). Thus, most likely these LDNs are not activated normal neutrophils. In addition, since SLE patients have chronic inflammation, it is unlikely that their LDNs present immunosuppressive activity. All these possibilities need to be further studied in the future. However, one serious limitation for the characterization of these cells is that there are not specific molecular markers that could distinguish among these possible neutrophils subpopulations.

#### Tumor-associated neutrophils (TANs)

**Figure 5:**
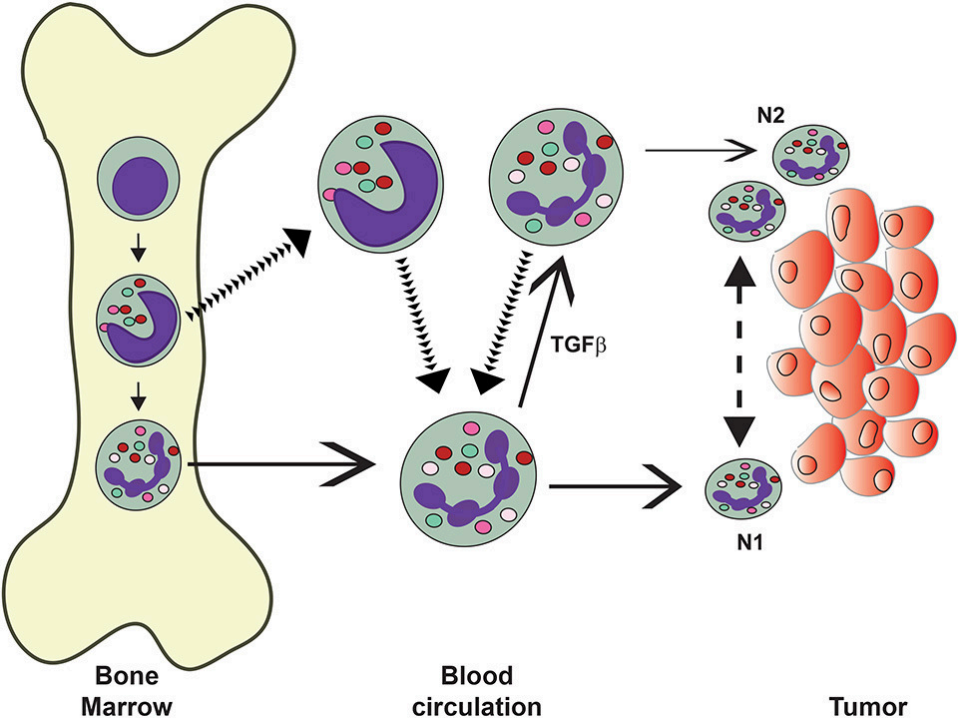
Neutrophils in the circulation display different phenotypes. Mature (normal) neutrophils (PMN) leave the bone marrow and display the classical pro-inflammatory and anti-tumor properties of these cells. It is thought that these PMN can migrate into tumors and display an anti-tumor (N1) phenotype. In tumor-bearing mice, immature neutrophils, such as band cells, also leave the bone marrow into the circulation. These “low-density” neutrophils include granulocytic myeloid derived suppressor cells (G-MDSC) and neutrophils with immunosuppressive properties. These cells can infiltrate tumors and display pro-tumor (N2) phenotype. Under the influence of transforming growth factor-beta (TGF-β, normal PMN can change into “low-density” neutrophils. The exact origin of recruited neutrophils is not known. Also, it is not clear if N1 cells can change into N2 cells and vice versa under the influence of the tumor microenvironment.

The phenotypic changes of circulating neutrophils during tumor progression are also related to infiltration of neutrophils into tumors. Unfortunately, the relationship among immunosuppressive cells (MDSC), LDNs, high-density (normal) neutrophils, and tumor-associated neutrophils (TANs) is just beginning to be elucidated (Mishalian et al., 2013; Uribe-Querol and Rosales, 2015; Figure 5).

Depending on the phenotype displayed by TANs in tumor-bearing mice, they have been classified as N1 or N2 (Fridlender et al., 2009). This classification is analogous to antitumor tumor-infiltrating macrophages (M1) or protumor macrophages (M2) (Galdiero et al., 2013). Murine N1 TANs are proinflammatory and antitumorigenic. In contrast, N2 TANs are protumorigenic (Fridlender et al., 2009). When tumor-bearing mice were treated to inhibit transforming growth factor-beta (TGF-β functions, the CD11b^+^ Ly6G^+^ neutrophils recruited to tumors were hypersegmented, more cytotoxic and more proinflammatory (N1). In contrast, in presence of TGF-β, had a protumor (N2) phenotype. Therefore, it seems that TGF-β within the tumor microenvironment induces a population of TANs with a protumor activity (Fridlender et al., 2009). This means that TANs can display an antitumor (N1) phenotype or a pro-tumor (N2) phenotype depending on the tumor microenvironment (Sionov et al., 2015). In addition, in tumor-bearing animals the LDNs increased progressively in circulation, were not cytotoxic, and had reduced expression of cytokines (Sagiv et al., 2015). The authors proposed that these immunosuppressive LDNs (G-MDSC) are the source of N2 TANs (Sagiv et al., 2015) (Figure 5). This is consistent with the idea that some of the LDNs are indeed immature neutrophils (Sagiv et al., 2015). Also, in the same murine model mature neutrophils were capable of becoming LDNs upon treatment with TGF-β (Sagiv et al., 2015). This has been interpreted as normal mature neutrophils having the capacity to infiltrate tumors and being cytotoxic, but under the influence of TGF-β, being able to change into N2 cells (Mishalian et al., 2013) (Figure 5). Although attractive, this hypothesis requires further evidence to be validated, since TGF-β was able to induce this change in tumor-bearing mice, but it had no effect on tumor-free mice (Sagiv et al., 2015).

It is important to emphasize here that the paradigm of N1 and N2 TANs has been described only in murine models of cancer, and that the nature and function of TANs in the tumor microenvironment remains largely unknown, particularly with human tumors. There are only two reports on isolation of human TANs, but they describe controversial data on their immunosuppression capacity. In one report, TANs were isolated from digested human lung tumors (Eruslanov et al., 2014). These TANs had an activated phenotype (CD62L^low^ CD54^hi^) and produced l proinflammatory cytokines. In consequence, these TANs stimulated T cell proliferation and interferon gamma (IFN-γ) production (Eruslanov et al., 2014). In another report, TANs were isolated from a colorectal tumor and found to present a typical neutrophil morphology. These TANs had the phenotype CD45^+^ Lin^−^ HLADR^−^ CD11b^+^ CD33^+^ CD66b^+^, and were classified as G-MDSC (Wu et al., 2014). These TANs secreted arginase 1 (ARG1) and ROS, and inhibited proliferation of activated autologous T cells and IFN-γ production (Wu et al., 2014). Together these reports suggest that neutrophils are present in three subpopulations in cancer: normal high-density neutrophils, immature LDNs (G-MDSC), and large mature LDNs (Figure 5). Early TANs (N1) are not immunosuppressive, but rather stimulate T cell responses (Eruslanov et al., 2014). Latter, the cells acquire an N2 phenotype and become immunosuppressive (Wu et al., 2014) (Figure 5).

#### Immunogenic cell death

Recently, another important role of neutrophils in anticancer therapy has been described. Immunogenic cell death can stimulate neutrophils to utilize cytotoxicity against residual live cancer cells after therapy. The concept of immunosurveillance explains how only the most immunoevasive or highly mutagenic neoplastic cells are able to generate clinically relevant tumors (Dunn et al., 2002; Schreiber et al., 2011). Yet, the immune system is capable of recognizing altered-self molecules in damaged cells through endogenously derived danger signals or alarmins (Bianchi, 2007; Garg et al., 2014). As a consequence of cell death, alarmins, collectively referred to as “damage-associated molecular patterns” (DAMPs), enhance sensing of dying cells by innate immune cells (Zitvogel et al., 2010; Garg et al., 2015b). Release of DAMPs can either be achieved in an unregulated fashion by necrosis (Aaes et al., 2016) or in a regulated fashion by immunogenic cell death, also called immunogenic apoptosis (Kepp et al., 2014; Garg et al., 2015a). Anti-cancer therapy-induced cancer cell death can be subdivided into three distinct types i.e., tolerogenic cell death, inflammatory cell death, and immunogenic cell death. The details of these types of cell death are beyond the scope of the present publication, but the reader is directed to some excellent recent reviews (Green et al., 2009; Garg et al., 2016; Garg and Agostinis, 2017). Briefly, immunogenic cell death is characterized by a defined discharge of DAMPs, type I interferon response, and the production of pathogen response-like chemokines (CXCL1, CCL2, and CXCL10) (Garg et al., 2017b) that together raise the immunogenic potential of dying cancer cells.

Through immunogenic cell death, after some types of anti-cancer therapy (Galluzzi et al., 2012; Bezu et al., 2015; Garg et al., 2017a), a damaged cancer cell produces specific inflammatory chemokines to recruit neutrophils as first innate immune cells (Kolaczkowska and Kubes, 2013; Garg et al., 2017b). The damaged cancer cells also express two important “eat me” signals on their membranes, namely, phosphatidylserine, and calreticulin. These signals trigger neutrophil phagocytosis of cancer cells and pro-inflammatory stimulation, leading to a change in neutrophil phenotype (Garg et al., 2017b). Neutrophils expressed a mature phenotype characterized by expression of CD86^hi^ MHC-II^hi^, and an activated phenotype characterized by IL-6^hi^ IL-1β^hi^ IL-10^low^ expression (Garg et al., 2017b). As a result, neutrophils stimulated by immunogenic cell death showed cytotoxicity against residual live cancer cells (Garg et al., 2017b). Thus, neutrophils interacting with immunogenic apoptotic cells gain a pro-inflammatory profile, culminating into neutrophil dependent cytotoxicity against residual cancer cells.

## Conclusion

There is a lot of excitement around the many “new” neutrophil functions just discovered recently. The realization that neutrophils do in fact perform many more functions than just antimicrobial responses, and the fact that neutrophils with different phenotypes have been reported in various tissues and pathological conditions, suggest that indeed different neutrophils exist. However, in most reports the evidence is circumstantial and we are in need for solid experimental proof that the cells described are in fact novel neutrophil subsets. What we have learned for sure so far is that in various pathological conditions, particularly cancer, distinct populations of mature and immature neutrophils are found in circulation. After gradient density centrifugation of blood, the mature high-density (or more properly normal-density) neutrophils mostly represent cells with a pro-inflammatory phenotype, while the LDNs are comprised of immature neutrophils and “activated” mature neutrophils. These cells in turn may migrate to tumors and become, at least in mice, N1 and N2 TANs (Figure 5). However, we have to highlight that the actual cell type responsible for the immunosuppressive properties of MDSC remains a mystery.

Many questions remain open, but at least two topics seem to be relevant at the moment. One important topic that needs to be addressed is whether mature neutrophils in circulation can be reprogrammed by external stimuli, or whether defined phenotypes are programmed in the bone marrow and neutrophils exit with a particular phenotypic signature. Evidence suggests that neutrophils are very plastic cells and in consequence the various subtypes described seem to be acquired in the tissues. However, these possibilities need to be formally tested. Another relevant topic is that the many functions described have not been assigned to particular phenotypes of neutrophils. This remains a complex issue, as there are currently no appropriate molecular markers to readily identify these different neutrophil subpopulations. This confusing scenario is the fuel for new and even more exciting research. We expect to learn new tricks from our favorite cell type in the near future.

## Author contributions

The author confirms being the sole contributor of this work and approved it for publication.

### Conflict of interest statement

The author declares that the research was conducted in the absence of any commercial or financial relationships that could be construed as a potential conflict of interest.

## References

[B1] Aaes T. L. Kaczmarek A. Delvaeye T. De Craene B. De Koker S. Heyndrickx L.. (2016). Vaccination with necroptotic cancer cells induces efficient anti-tumor immunity. Cell Rep. 15, 274–287. 10.1016/j.celrep.2016.03.037 27050509

[B2] Adrover J. M. Nicolás-Ávila J. A. Hidalgo A. (2016). Aging: a temporal dimension for neutrophils. Trends Immunol. 37, 334–345. 10.1016/j.it.2016.03.005 27083489

[B3] Beauvillain C. Cunin P. Doni A. Scotet M. Jaillon S. Loiry M. L.. (2011). CCR7 is involved in the migration of neutrophils to lymph nodes. Blood 117, 1196–1204. 10.1182/blood-2009-11-254490 21051556

[B4] Berger-Achituv S. Brinkmann V. Abed U. A. Kühn L. I. Ben-Ezra J. Elhasid R.. (2013). A proposed role for neutrophil extracellular traps in cancer immunoediting. Front. Immunol. 4:48. 10.3389/fimmu.2013.00048 23508552 PMC3589747

[B5] Beyrau M. Bodkin J. V. Nourshargh S. (2012). Neutrophil heterogeneity in health and disease: a revitalized avenue in inflammation and immunity. Open Biol. 2:120134. 10.1098/rsob.120134 23226600 PMC3513838

[B6] Bezu L. Gomes-de-Silva L. C. Dewitte H. Breckpot K. Fucikova J. Spisek R. (2015). Combinatorial strategies for the induction of immunogenic cell death. Front. Immunol. 6:187 10.3389/fimmu.2015.00187 25964783 PMC4408862

[B7] Bianchi M. E. (2007). DAMPs, PAMPs and alarmins: all we need to know about danger. J. Leukoc. Biol. 81, 1–5. 10.1189/jlb.0306164 17032697

[B8] Bonaventura A. Liberale L. Carbone F. Vecchié A. Diaz-Cañestro C. Camici G. G.. (2018). The pathophysiological role of neutrophil extracellular traps in inflammatory diseases. Thromb. Haemost. 118, 6–27. 10.1160/TH17-09-0630 29304522

[B9] Borregaard N. (2010). Neutrophils, from marrow to microbes. Immunity 33, 657–670. 10.1016/j.immuni.2010.11.011 21094463

[B10] Bournazou I. Pound J. D. Duffin R. Bournazos S. Melville L. A. Brown S. B.. (2009). Apoptotic human cells inhibit migration of granulocytes via release of lactoferrin. J. Clin. Invest. 119, 20–32. 10.1172/JCI36226 19033648 PMC2613454

[B11] Böyum A. (1968). Isolation of mononuclear cells and granulocytes from human blood. Isolation of monuclear cells by one centrifugation, and of granulocytes by combining centrifugation and sedimentation at 1 g. Scand, J. Clin. Lab. Invest. Suppl. 97, 77–89. 4179068

[B12] Braganza J. M. Lee S. H. McCloy R. F. McMahon M. J. (2011). Chronic pancreatitis. Lancet 377, 1184–1197. 10.1016/S0140-6736(10)61852-1 21397320

[B13] Brandau S. Moses K. Lang S. (2013). The kinship of neutrophils and granulocytic myeloid-derived suppressor cells in cancer: cousins, siblings or twins? Semin. Cancer Biol. 23, 171–182. 10.1016/j.semcancer.2013.02.007 23459190

[B14] Brandau S. Trellakis S. Bruderek K. Schmaltz D. Steller G. Elian M.. (2011). Myeloid-derived suppressor cells in the peripheral blood of cancer patients contain a subset of immature neutrophils with impaired migratory properties. J. Leukoc. Biol. 89, 311–317. 10.1189/jlb.0310162 21106641

[B15] Branzk N. Lubojemska A. Hardison S. E. Wang Q. Gutierrez M. G. Brown G. D.. (2014). Neutrophils sense microbe size and selectively release neutrophil extracellular traps in response to large pathogens. Nat. Immunol. 15, 1017–1025. 10.1038/ni.2987 25217981 PMC4236687

[B16] Bratton D. L. Henson P. M. (2011). Neutrophil clearance: when the party is over, clean-up begins. Trends Immunol. 32, 350–357. 10.1016/j.it.2011.04.009 21782511 PMC3151332

[B17] Brill A. Fuchs T. A. Savchenko A. S. Thomas G. M. Martinod K. De Meyer S. F.. (2012). Neutrophil extracellular traps promote deep vein thrombosis in mice. J. Thromb. Haemost. 10, 136–144. 10.1111/j.1538-7836.2011.04544.x 22044575 PMC3319651

[B18] Buckley C. D. Ross E. A. McGettrick H. M. Osborne C. E. Haworth O. Schmutz C.. (2006). Identification of a phenotypically and functionally distinct population of long-lived neutrophils in a model of reverse endothelial migration. J. Leukoc. Biol. 79, 303–311. 10.1189/jlb.0905496 16330528

[B19] Busso N. So A. (2012). Microcrystals as DAMPs and their role in joint inflammation. Rheumatology 51, 1154–1160. 10.1093/rheumatology/ker524 22332122

[B20] Carmona-Rivera C. Kaplan M. J. (2013). Low-density granulocytes: a distinct class of neutrophils in systemic autoimmunity. Semin. Immunopathol. 35, 455–463. 10.1007/s00281-013-0375-7 23553215 PMC4007274

[B21] Caruso R. A. Bellocco R. Pagano M. Bertoli G. Rigoli L. Inferrera C. (2002). Prognostic value of intratumoral neutrophils in advanced gastric carcinoma in a high-risk area in northern Italy. Mod. Pathol. 15, 831–837. 10.1097/01.MP.0000020391.98998.6B 12181268

[B22] Casanova-Acebes M. Pitaval C. Weiss L. A. Nombela-Arrieta C. Chèvre R. A-González N.. (2013). Rhythmic modulation of the hematopoietic niche through neutrophil clearance. Cell 153, 1025–1035. 10.1016/j.cell.2013.04.040 23706740 PMC4128329

[B23] Cerutti A. Puga I. Magri G. (2013). The B cell helper side of neutrophils. J. Leukoc. Biol. 94, 677–682. 10.1189/jlb.1112596 23630389 PMC3774846

[B24] Chavakis E. Choi E. Y. Chavakis T. (2009). Novel aspects in the regulation of the leukocyte adhesion cascade. Thromb. Haemost. 102, 191–197. 10.1160/TH08-12-0844 19652868 PMC2722029

[B25] Chen F. Wu W. Millman A. Craft J. F. Chen E. Patel N.. (2014). Neutrophils prime a long-lived effector macrophage phenotype that mediates accelerated helminth expulsion. Nat. Immunol. 15, 938–946. 10.1038/ni.2984 25173346 PMC4479254

[B26] Chistiakov D. A. Bobryshev Y. V. Orekhov A. N. (2015). Neutrophil's weapons in atherosclerosis. Exp. Mol. Pathol. 99, 663–671. 10.1016/j.yexmp.2015.11.011 26551083

[B27] Clark S. R. Ma A. C. Tavener S. A. McDonald B. Goodarzi Z. Kelly M. M.. (2007). Platelet TLR4 activates neutrophil extracellular traps to ensnare bacteria in septic blood. Nat. Med. 13, 463–469. 10.1038/nm1565 17384648

[B28] Clarke T. B. Davis K. M. Lysenko E. S. Zhou A. Y. Yu Y. Weiser J. N. (2010). Recognition of peptidoglycan from the microbiota by Nod1 enhances systemic innate immunity. Nat. Med. 16, 228–231. 10.1038/nm.2087 20081863 PMC4497535

[B29] Cloke T. Munder M. Taylor G. Müller I. Kropf P. (2012). Characterization of a novel population of low-density granulocytes associated with disease severity in HIV-1 infection. PLoS ONE 7:e48939. 10.1371/journal.pone.0048939 23152825 PMC3496742

[B30] Coffelt S. B. Wellenstein M. D. de Visser K. E. (2016). Neutrophils in cancer: neutral no more. Nat. Rev. Cancer 16, 431–446. 10.1038/nrc.2016.52 27282249

[B31] Daley J. M. Thomay A. A. Connolly M. D. Reichner J. S. Albina J. E. (2008). Use of Ly6G-specific monoclonal antibody to deplete neutrophils in mice. J. Leukoc. Biol. 83, 64–70. 10.1189/jlb.0407247 17884993

[B32] Damuzzo V. Pinton L. Desantis G. Solito S. Marigo I. Bronte V.. (2015). Complexity and challenges in defining myeloid-derived suppressor cells. Cytometry B Clin. Cytom. 88, 77–91. 10.1002/cytob.21206 25504825 PMC4405078

[B33] Dancey J. T. Deubelbeiss K. A. Harker L. A. Finch C. A. (1976). Neutrophil kinetics in man. J. Clin. Invest. 58, 705–715. 10.1172/JCI108517 956397 PMC333229

[B34] Deng Y. Ye J. Luo Q. Huang Z. Peng Y. Xiong G.. (2016). Low-density granulocytes are elevated in mycobacterial infection and associated with the severity of tuberculosis. PLoS ONE 11:e0153567. 10.1371/journal.pone.0153567 27073889 PMC4830625

[B35] Denny M. F. Yalavarthi S. Zhao W. Thacker S. G. Anderson M. Sandy A. R.. (2010). A distinct subset of proinflammatory neutrophils isolated from patients with systemic lupus erythematosus induces vascular damage and synthesizes type I IFNs. J. Immunol. 184, 3284–3297. 10.4049/jimmunol.0902199 20164424 PMC2929645

[B36] Deshmukh H. S. Liu Y. Menkiti O. R. Mei J. Dai N. O'Leary C. E.. (2014). The microbiota regulates neutrophil homeostasis and host resistance to *Escherichia coli* K1 sepsis in neonatal mice. Nat. Med. 20, 524–530. 10.1038/nm.3542 24747744 PMC4016187

[B37] Devi S. Wang Y. Chew W. K. Lima R. A.-González N. Mattar C. N.. (2013). Neutrophil mobilization via plerixafor-mediated CXCR4 inhibition arises from lung demargination and blockade of neutrophil homing to the bone marrow. J. Exp. Med. 210, 2321–2336. 10.1084/jem.20130056 24081949 PMC3804935

[B38] Di Nisio M. van Es N. Büller H. R. (2016). Deep vein thrombosis and pulmonary embolism. Lancet 388, 3060–3073. 10.1016/S0140-6736(16)30514-1 27375038

[B39] Döring Y. Drechsler M. Wantha S. Kemmerich K. Lievens D. Vijayan S.. (2012). Lack of neutrophil-derived CRAMP reduces atherosclerosis in mice. Circ. Res. 110, 1052–1056. 10.1161/CIRCRESAHA.112.265868 22394519

[B40] Döring Y. Soehnlein O. Weber C. (2017). Neutrophil extracellular traps in atherosclerosis and atherothrombosis. Circ. Res. 120, 736–743. 10.1161/CIRCRESAHA.116.309692 28209798

[B41] Downey G. P. Fukushima T. Fialkow L. Waddell T. K. (1995). Intracellular signaling in neutrophil priming and activation. Semin. Cell Biol. 6, 345–356. 10.1016/S1043-4682(05)80005-4 8748142

[B42] Drechsler M. Megens R. T. van Zandvoort M. Weber C. Soehnlein O. (2010). Hyperlipidemia-triggered neutrophilia promotes early atherosclerosis. Circulation 122, 1837–1845. 10.1161/CIRCULATIONAHA.110.961714 20956207

[B43] Duffy D. Perrin H. Abadie V. Benhabiles N. Boissonnas A. Liard C.. (2012). Neutrophils transport antigen from the dermis to the bone marrow, initiating a source of memory CD8+ T cells. Immunity 37, 917–929. 10.1016/j.immuni.2012.07.015 23142782

[B44] Dumitru C. A. Moses K. Trellakis S. Lang S. Brandau S. (2012). Neutrophils and granulocytic myeloid-derived suppressor cells: immunophenotyping, cell biology and clinical relevance in human oncology. Cancer Immunol. Immunother. 61, 1155–1167. 10.1007/s00262-012-1294-5 22692756 PMC11028504

[B45] Dunn G. P. Bruce A. T. Ikeda H. Old L. J. Schreiber R. D. (2002). Cancer immunoediting: from immunosurveillance to tumor escape. Nat. Immunol. 3, 991–998. 10.1038/ni1102-991 12407406

[B46] Eash K. J. Greenbaum A. M. Gopalan P. K. Link D. C. (2010). CXCR2 and CXCR4 antagonistically regulate neutrophil trafficking from murine bone marrow. J. Clin. Invest. 120, 2423–2431. 10.1172/JCI41649 20516641 PMC2898597

[B47] Eash K. J. Means J. M. White D. W. Link D. C. (2009). CXCR4 is a key regulator of neutrophil release from the bone marrow under basal and stress granulopoiesis conditions. Blood 113, 4711–4719. 10.1182/blood-2008-09-177287 19264920 PMC2680371

[B48] El-Benna J. Hurtado-Nedelec M. Marzaioli V. Marie J. C. Gougerot-Pocidalo M. A. Dang P. M. (2016). Priming of the neutrophil respiratory burst: role in host defense and inflammation. Immunol. Rev. 273, 180–193. 10.1111/imr.12447 27558335

[B49] Elliott M. R. Chekeni F. B. Trampont P. C. Lazarowski E. R. Kadl A. Walk S. F.. (2009). Nucleotides released by apoptotic cells act as a find-me signal to promote phagocytic clearance. Nature 461, 282–286. 10.1038/nature08296 19741708 PMC2851546

[B50] Ericson J. A. Duffau P. Yasuda K. Ortiz-Lopez A. Rothamel K. Rifkin I. R.. (2014). Gene expression during the generation and activation of mouse neutrophils: implication of novel functional and regulatory pathways. PLoS ONE 9:e108553. 10.1371/journal.pone.0108553 25279834 PMC4184787

[B51] Eruslanov E. B. Bhojnagarwala P. S. Quatromoni J. G. Stephen T. L. Ranganathan A. Deshpande C.. (2014). Tumor-associated neutrophils stimulate T cell responses in early-stage human lung cancer. J. Clin. Invest. 124, 5466–5480. 10.1172/JCI77053 25384214 PMC4348966

[B52] Eskan M. A. Jotwani R. Abe T. Chmelar J. Lim J. H. Liang S.. (2012). The leukocyte integrin antagonist Del-1 inhibits IL-17-mediated inflammatory bone loss. Nat. Immunol. 13, 465–473. 10.1038/ni.2260 22447028 PMC3330141

[B53] Etulain J. Martinod K. Wong S. L. Cifuni S. M. Schattner M. Wagner D. D. (2015). P-selectin promotes neutrophil extracellular trap formation in mice. Blood 126, 242–246. 10.1182/blood-2015-01-624023 25979951 PMC4497964

[B54] Faria S. S. Fernandes P. C. Jr. Silva M. J. Lima V. C. Fontes W. Freitas-Junior R.. (2016). The neutrophil-to-lymphocyte ratio: a narrative review. Ecancermedicalscience 10:702. 10.3332/ecancer.2016.702 28105073 PMC5221645

[B55] Favaloro J. Liyadipitiya T. Brown R. Yang S. Suen H. Woodland N.. (2014). Myeloid derived suppressor cells are numerically, functionally and phenotypically different in patients with multiple myeloma. Leuk. Lymphoma. 55, 2893–2900. 10.3109/10428194.2014.904511 24625328

[B56] Fortunati E. Kazemier K. M. Grutters J. C. Koenderman L. Van den Bosch v. J. (2009). Human neutrophils switch to an activated phenotype after homing to the lung irrespective of inflammatory disease. Clin. Exp. Immunol. 155, 559–566. 10.1111/j.1365-2249.2008.03791.x 19077082 PMC2669533

[B57] Fridlender Z. G. Albelda S. M. (2012). Tumor-associated neutrophils: friend or foe? Carcinogenesis 33, 949–955. 10.1093/carcin/bgs123 22425643

[B58] Fridlender Z. G. Sun J. Kim S. Kapoor V. Cheng G. Ling L.. (2009). Polarization of tumor-associated neutrophil phenotype by TGF-beta, “N1” versus “N2” TAN. Cancer Cell 16, 183–194. 10.1016/j.ccr.2009.06.017 19732719 PMC2754404

[B59] Fu J. Tobin M. C. Thomas L. L. (2014). Neutrophil-like low-density granulocytes are elevated in patients with moderate to severe persistent asthma. Ann. Allergy Asthma Immunol. 113, 635–640. 10.1016/j.anai.2014.08.024 25256681

[B60] Fuchs T. A. Abed U. Goosmann C. Hurwitz R. Schulze I. Wahn V.. (2007). Novel cell death program leads to neutrophil extracellular traps. J. Cell Biol. 176, 231–241. 10.1083/jcb.200606027 17210947 PMC2063942

[B61] Fuchs T. A. Brill A. Duerschmied D. Schatzberg D. Monestier M. Myers D. D.. (2010). Extracellular DNA traps promote thrombosis. Proc. Natl. Acad. Sci. U.S.A. 107, 15880–15885. 10.1073/pnas.1005743107 20798043 PMC2936604

[B62] Gaffen S. L. Jain R. Garg A. V. Cua D. J. (2014). The IL-23-IL-17 immune axis: from mechanisms to therapeutic testing. Nat. Rev. Immunol. 14, 585–600. 10.1038/nri3707 25145755 PMC4281037

[B63] Galdiero M. R. Garlanda C. Jaillon S. Marone G. Mantovani A. (2013). Tumor associated macrophages and neutrophils in tumor progression. J. Cell. Physiol. 228, 1404–1412. 10.1002/jcp.24260 23065796

[B64] Galluzzi L. Senovilla L. Zitvogel L. Kroemer G. (2012). The secret ally: immunostimulation by anticancer drugs. Nat. Rev. Drug Discov. 11, 215–233. 10.1038/nrd3626 22301798

[B65] García-García E. Uribe-Querol E. Rosales C. (2013). A simple and efficient method to detect nuclear factor activation in human neutrophils by flow cytometry. J. Vis. Exp. 74:e50410 10.3791/50410 PMC3653551 23603868

[B66] Garcia-Romo G. S. Caielli S. Vega B. Connolly J. Allantaz F. Xu Z.. (2011). Netting neutrophils are major inducers of type I IFN production in pediatric systemic lupus erythematosus. Sci. Transl. Med. 3:73ra20. 10.1126/scitranslmed.3001201 21389264 PMC3143837

[B67] Garg A. D. Agostinis P. (2017). Cell death and immunity in cancer: from danger signals to mimicry of pathogen defense responses. Immunol. Rev. 280, 126–148. 10.1111/imr.12574 29027218

[B68] Garg A. D. Dudek-Peric A. M. Romano E. Agostinis P. (2015a). Immunogenic cell death. Int. J. Dev. Biol. 59, 131–140. 10.1387/ijdb.150061pa 26374534

[B69] Garg A. D. Galluzzi L. Apetoh L. Baert T. Birge R. B. Bravo-San Pedro J. M.. (2015b). Molecular and translational classifications of DAMPs in immunogenic cell death. Front. Immunol. 6:588. 10.3389/fimmu.2015.00588 26635802 PMC4653610

[B70] Garg A. D. Martin S. Golab J. Agostinis P. (2014). Danger signalling during cancer cell death: origins, plasticity and regulation. Cell Death Differ. 21, 26–38. 10.1038/cdd.2013.48 23686135 PMC3858605

[B71] Garg A. D. More S. Rufo N. Mece O. Sassano M. L. Agostinis P.. (2017a). Trial watch: immunogenic cell death induction by anticancer chemotherapeutics. Oncoimmunology 6:e1386829. 10.1080/2162402X.2017.1386829 29209573 PMC5706600

[B72] Garg A. D. Romano E. Rufo N. Agostinis P. (2016). Immunogenic versus tolerogenic phagocytosis during anticancer therapy: mechanisms and clinical translation. Cell Death Differ. 23, 938–951. 10.1038/cdd.2016.5 26891691 PMC4987738

[B73] Garg A. D. Vandenberk L. Fang S. Fasche T. Van Eygen S. Maes J.. (2017b). Pathogen response-like recruitment and activation of neutrophils by sterile immunogenic dying cells drives neutrophil-mediated residual cell killing. Cell Death Differ. 24, 832–843. 10.1038/cdd.2017.15 28234357 PMC5423108

[B74] Garley M. Jabłonska E. Dabrowska D. (2016). NETs in cancer. Tumour Biol. 37, 14355–14361. 10.1007/s13277-016-5328-z 27614687

[B75] Gisterå A. Hansson G. K. (2017). The immunology of atherosclerosis. Nat. Rev. Nephrol. 13, 368–380. 10.1038/nrneph.2017.51 28392564

[B76] Gordy C. Pua H. Sempowski G. D. He Y. W. (2011). Regulation of steady-state neutrophil homeostasis by macrophages. Blood 117, 618–629. 10.1182/blood-2010-01-265959 20980680 PMC3031484

[B77] Görgens A. Radtke S. Möllmann M. Cross M. Dürig J. Horn P. A.. (2013). Revision of the human hematopoietic tree: granulocyte subtypes derive from distinct hematopoietic lineages. Cell Rep. 3, 1539–1552. 10.1016/j.celrep.2013.04.025 23707063

[B78] Gorlino C. V. Ranocchia R. P. Harman M. F. García I. A. Crespo M. I. Morón G.. (2014). Neutrophils exhibit differential requirements for homing molecules in their lymphatic and blood trafficking into draining lymph nodes. J. Immunol. 193, 1966–1974. 10.4049/jimmunol.1301791 25015824

[B79] Grayson P. C. Carmona-Rivera C. Xu L. Lim N. Gao Z. Asare A. L.. (2015). Neutrophil-related gene expression and low-density granulocytes associated with disease activity and response to treatment in antineutrophil cytoplasmic antibody-associated vasculitis. Arthritis Rheumatol. 67, 1922–1932. 10.1002/art.39153 25891759 PMC4485551

[B80] Green D. R. Ferguson T. Zitvogel L. Kroemer G. (2009). Immunogenic and tolerogenic cell death. Nat. Rev. Immunol. 9, 353–363. 10.1038/nri2545 19365408 PMC2818721

[B81] Greenlee-Wacker M. C. (2016). Clearance of apoptotic neutrophils and resolution of inflammation. Immunol. Rev. 273, 357–370. 10.1111/imr.12453 27558346 PMC5000862

[B82] Gukovskaya A. S. Pandol S. J. Gukovsky I. (2016). New insights into the pathways initiating and driving pancreatitis. Curr. Opin. Gastroenterol. 32, 429–435. 10.1097/MOG.0000000000000301 PMC5235997 27428704

[B83] Gukovskaya A. S. Vaquero E. Zaninovic V. Gorelick F. S. Lusis A. J. Brennan M. L.. (2002). Neutrophils and NADPH oxidase mediate intrapancreatic trypsin activation in murine experimental acute pancreatitis. Gastroenterology 122, 974–984. 10.1053/gast.2002.32409 11910350

[B84] Guthrie G. J. Charles K. A. Roxburgh C. S. Horgan P. G. McMillan D. C. Clarke S. J. (2013). The systemic inflammation-based neutrophil-lymphocyte ratio: experience in patients with cancer. Crit. Rev. Oncol. Hematol. 88, 218–230. 10.1016/j.critrevonc.2013.03.010 23602134

[B85] Hacbarth E. Kajdacsy-Balla A. (1986). Low density neutrophils in patients with systemic lupus erythematosus, rheumatoid arthritis, and acute rheumatic fever. Arthritis Rheum. 29, 1334–1342. 10.1002/art.1780291105 2430586

[B86] Häger M. Cowland J. B. Borregaard N. (2010). Neutrophil granules in health and disease. J. Intern. Med. 268, 25–34. 10.1111/j.1365-2796.2010.02237.x 20497300

[B87] Hahn J. Knopf J. Maueröder C. Kienhöfer D. Leppkes M. Herrmann M. (2016). Neutrophils and neutrophil extracellular traps orchestrate initiation and resolution of inflammation. Clin. Exp. Rheumatol. 34, 6–8. 27586795

[B88] Hajishengallis G. Chavakis T. (2013). Endogenous modulators of inflammatory cell recruitment. Trends Immunol. 34, 1–6. 10.1016/j.it.2012.08.003 22951309 PMC3703146

[B89] Halangk W. Lerch M. M. Brandt-Nedelev B. Roth W. Ruthenbuerger M. Reinheckel T.. (2000). Role of cathepsin B in intracellular trypsinogen activation and the onset of acute pancreatitis. J. Clin. Invest. 106, 773–781. 10.1172/JCI9411 10995788 PMC381392

[B90] Hampton H. R. Chtanova T. (2016). The lymph node neutrophil. Semin. Immunol. 28, 129–136. 10.1016/j.smim.2016.03.008 27025975

[B91] Hansson G. K. Libby P. Tabas I. (2015). Inflammation and plaque vulnerability. J. Intern. Med. 278, 483–493. 10.1111/joim.12406 26260307 PMC5082111

[B92] Hergott C. B. Roche A. M. Tamashiro E. Clarke T. B. Bailey A. G. Laughlin A.. (2016). Peptidoglycan from the gut microbiota governs the lifespan of circulating phagocytes at homeostasis. Blood 127, 2460–2471. 10.1182/blood-2015-10-675173 26989200 PMC4874226

[B93] Jablonska J. Leschner S. Westphal K. Lienenklaus S. Weiss S. (2010). Neutrophils responsive to endogenous IFN-beta regulate tumor angiogenesis and growth in a mouse tumor model. J. Clin. Invest. 120, 1151–1164. 10.1172/JCI37223 20237412 PMC2846036

[B94] Jaiswal S. Jamieson C. H. Pang W. W. Park C. Y. Chao M. P. Majeti R.. (2009). CD47 is upregulated on circulating hematopoietic stem cells and leukemia cells to avoid phagocytosis. Cell 138, 271–285. 10.1016/j.cell.2009.05.046 19632178 PMC2775564

[B95] Jiao J. Dragomir A. C. Kocabayoglu P. Rahman A. H. Chow A. Hashimoto D.. (2014). Central role of conventional dendritic cells in regulation of bone marrow release and survival of neutrophils. J. Immunol. 192, 3374–3382. 10.4049/jimmunol.1300237 24591364 PMC4144807

[B96] Kanamaru R. Ohzawa H. Miyato H. Matsumoto S. Haruta H. Kurashina K.. (2018). Low density neutrophils (LDN) in postoperative abdominal cavity assist the peritoneal recurrence through the production of neutrophil extracellular traps (NETs). Sci. Rep. 8:632. 10.1038/s41598-017-19091-2 29330531 PMC5766579

[B97] Kepp O. Senovilla L. Vitale I. Vacchelli E. Adjemian S. Agostinis P.. (2014). Consensus guidelines for the detection of immunogenic cell death. Oncoimmunology 3:e955691. 10.4161/21624011.2014.955691 25941621 PMC4292729

[B98] Keskinov A. A. Shurin M. R. (2015). Myeloid regulatory cells in tumor spreading and metastasis. Immunobiology 220, 236–242. 10.1016/j.imbio.2014.07.017 25178934

[B99] Kessenbrock K. Krumbholz M. Schönermarck U. Back W. Gross W. L. Werb Z.. (2009). Netting neutrophils in autoimmune small-vessel vasculitis. Nat. Med. 15, 623–625. 10.1038/nm.1959 19448636 PMC2760083

[B100] Khandpur R. Carmona-Rivera C. Vivekanandan-Giri A. Gizinski A. Yalavarthi S. Knight J. S.. (2013). NETs are a source of citrullinated autoantigens and stimulate inflammatory responses in rheumatoid arthritis. Sci. Transl. Med. 5:178ra40. 10.1126/scitranslmed.3005580 23536012 PMC3727661

[B101] Kim H. K. De La Luz Sierra M. Williams C. K. Gulino A. V. Tosato G. (2006). G-CSF down-regulation of CXCR4 expression identified as a mechanism for mobilization of myeloid cells. Blood 108, 812–820. 10.1182/blood-2005-10-4162 16537807 PMC1895847

[B102] Kingsbury S. R. Conaghan P. G. McDermott M. F. (2011). The role of the NLRP3 inflammasome in gout. J. Inflamm. Res. 4, 39–49. 10.2147/JIR.S11330 22096368 PMC3218743

[B103] Knight J. S. Luo W. O'Dell A. A. Yalavarthi S. Zhao W. Subramanian V.. (2014). Peptidylarginine deiminase inhibition reduces vascular damage and modulates innate immune responses in murine models of atherosclerosis. Circ. Res. 114, 947–956. 10.1161/CIRCRESAHA.114.303312 24425713 PMC4185401

[B104] Köhler A. De Filippo K. Hasenberg M. van den Brandt C. Nye E. Hosking M. P.. (2011). G-CSF-mediated thrombopoietin release triggers neutrophil motility and mobilization from bone marrow via induction of CXCR2 ligands. Blood 117, 4349–4357. 10.1182/blood-2010-09-308387 21224471 PMC3087483

[B105] Kolaczkowska E. Kubes P. (2013). Neutrophil recruitment and function in health and inflammation. Nat. Rev. Immunol. 13, 159–175. 10.1038/nri3399 23435331

[B106] Kraakman M. J. Lee M. K. Al-Sharea A. Dragoljevic D. Barrett T. J. Montenont E.. (2017). Neutrophil-derived S100 calcium-binding proteins A8/A9 promote reticulated thrombocytosis and atherogenesis in diabetes. J. Clin. Invest. 127, 2133–2147. 10.1172/JCI92450 28504650 PMC5451242

[B107] Kropf P. Baud D. Marshall S. E. Munder M. Mosley A. Fuentes J. M.. (2007). Arginase activity mediates reversible T cell hyporesponsiveness in human pregnancy. Eur. J. Immunol. 37, 935–945. 10.1002/eji.200636542 17330821 PMC2699382

[B108] Lankisch P. G. Apte M. Banks P. A. (2015). Acute pancreatitis. Lancet 386, 85–96. 10.1016/S0140-6736(14)60649-8 25616312

[B109] Lechner M. G. Liebertz D. J. Epstein A. L. (2010). Characterization of cytokine-induced myeloid-derived suppressor cells from normal human peripheral blood mononuclear cells. J. Immunol. 185, 2273–2284. 10.4049/jimmunol.1000901 20644162 PMC2923483

[B110] Leppkes M. Maueröder C. Hirth S. Nowecki S. Günther C. Billmeier U.. (2016). Externalized decondensed neutrophil chromatin occludes pancreatic ducts and drives pancreatitis. Nat. Commun. 7:10973. 10.1038/ncomms10973 26964500 PMC4793047

[B111] Ley K. Laudanna C. Cybulsky M. I. Nourshargh S. (2007). Getting to the site of inflammation: the leukocyte adhesion cascade updated. Nat. Rev. Immunol. 7, 678–689. 10.1038/nri2156 17717539

[B112] Lin A. M. Rubin C. J. Khandpur R. Wang J. Y. Riblett M. Yalavarthi S.. (2011). Mast cells and neutrophils release IL-17 through extracellular trap formation in psoriasis. J. Immunol. 187, 490–500. 10.4049/jimmunol.1100123 21606249 PMC3119764

[B113] Mandruzzato S. Brandau S. Britten C. M. Bronte V. Damuzzo V. Gouttefangeas C.. (2016). Toward harmonized phenotyping of human myeloid-derived suppressor cells by flow cytometry: results from an interim study. Cancer. Immunol. Immunother. 65, 161–169. 10.1007/s00262-015-1782-5 26728481 PMC4726716

[B114] Manfredi A. A. Covino C. Rovere-Querini P. Maugeri N. (2015). Instructive influences of phagocytic clearance of dying cells on neutrophil extracellular trap generation. Clin. Exp. Immunol. 179, 24–29. 10.1111/cei.12320 24611549 PMC4260893

[B115] Manohar M. Verma A. K. Venkateshaiah S. U. Sanders N. L. Mishra A. (2017). Pathogenic mechanisms of pancreatitis. World J. Gastrointest. Pharmacol. Ther. 8, 10–25. 10.4292/wjgpt.v8.i1.10 28217371 PMC5292603

[B116] Mansuy-Aubert V. Zhou Q. L. Xie X. Gong Z. Huang J. Y. Khan A. R.. (2013). Imbalance between neutrophil elastase and its inhibitor α1-antitrypsin in obesity alters insulin sensitivity, inflammation, and energy expenditure. Cell Metab. 17, 534–548. 10.1016/j.cmet.2013.03.005 23562077 PMC3646573

[B117] Martin C. Burdon P. C. Bridger G. Gutierrez-Ramos J. C. Williams T. J. Rankin S. M. (2003). Chemokines acting via CXCR2 and CXCR4 control the release of neutrophils from the bone marrow and their return following senescence. Immunity 19, 583–593. 10.1016/S1074-7613(03)00263-2 14563322

[B118] Massberg S. Grahl L. von Bruehl M. L. Manukyan D. Pfeiler S. Goosmann C.. (2010). Reciprocal coupling of coagulation and innate immunity via neutrophil serine proteases. Nat. Med. 16, 887–896. 10.1038/nm.2184 20676107

[B119] Massena S. Christoffersson G. Vågesjö E. Seignez C. Gustafsson K. Binet F.. (2015). Identification and characterization of VEGF-A-responsive neutrophils expressing CD49d, VEGFR1, and CXCR4 in mice and humans. Blood 126, 2016–2026. 10.1182/blood-2015-03-631572 26286848 PMC4616235

[B120] Mathias J. R. Perrin B. J. Liu T. X. Kanki J. Look A. T. Huttenlocher A. (2012). Resolution of inflammation by retrograde chemotaxis of neutrophils in transgenic zebrafish. J. Leukoc. Biol. 80, 1281–1288. 10.1189/jlb.0506346 16963624

[B121] Maueröder C. Kienhöfer D. Hahn J. Schauer C. Manger B. Schett G.. (2015). How neutrophil extracellular traps orchestrate the local immune response in gout. J. Mol. Med. 93, 727–734. 10.1007/s00109-015-1295-x 26002146

[B122] Mayadas T. N. Cullere X. Lowell C. A. (2014). The multifaceted functions of neutrophils. Annu. Rev. Pathol. 9, 181–218. 10.1146/annurev-pathol-020712-164023 24050624 PMC4277181

[B123] Mazor R. Shurtz-Swirski R. Farah R. Kristal B. Shapiro G. Dorlechter F.. (2008). Primed polymorphonuclear leukocytes constitute a possible link between inflammation and oxidative stress in hyperlipidemic patients. Atherosclerosis 197, 937–943. 10.1016/j.atherosclerosis.2007.08.014 17869258

[B124] McGary C. T. Miele M. E. Welch D. R. (1995). Highly metastatic 13762NF rat mammary adenocarcinoma cell clones stimulate bone marrow by secretion of granulocyte-macrophage colony-stimulating factor/interleukin-3 activity. Am. J. Pathol. 147, 1668–1681. 7495292 PMC1869940

[B125] Merza M. Hartman H. Rahman M. Hwaiz R. Zhang E. Renström E.. (2015). Neutrophil extracellular traps induce trypsin activation, inflammation, and tissue damage in mice with severe acute pancreatitis. Gastroenterology 149, 1920–1931. 10.1053/j.gastro.2015.08.026 26302488

[B126] Michels A. Albánez S. Mewburn J. Nesbitt K. Gould T. J. Liaw P. C.. (2016). Histones link inflammation and thrombosis through the induction of Weibel-Palade body exocytosis. J. Thromb. Haemost. 14, 2274–2286. 10.1111/jth.13493 27589692

[B127] Mishalian I. Bayuh R. Levy L. Zolotarov L. Michaeli J. Fridlender Z. G. (2013). Tumor-associated neutrophils (TAN) develop pro-tumorigenic properties during tumor progression. Cancer Immunol. Immunother. 62, 1745–1756. 10.1007/s00262-013-1476-9 24092389 PMC11028422

[B128] Mishalian I. Granot Z. Fridlender Z. G. (2017). The diversity of circulating neutrophils in cancer. Immunobiology 222, 82–88. 10.1016/j.imbio.2016.02.001 26874580

[B129] Morisaki T. Goya T. Ishimitsu T. Torisu M. (1992). The increase of low density subpopulations and CD10 (CALLA) negative neutrophils in severely infected patients. Surg. Today. 22, 322–327. 10.1007/BF00308740 1392343

[B130] Nagaraj S. Schrum A. G. Cho H. I. Celis E. Gabrilovich D. (2010). Mechanism of T cell tolerance induced by myeloid-derived suppressor cells. J. Immunol. 184, 3106–3116. 10.4049/jimmunol.0902661 20142361 PMC2832724

[B131] Nagase H. Miyamasu M. Yamaguchi M. Imanishi M. Tsuno N. H. Matsushima K.. (2002). Cytokine-mediated regulation of CXCR4 expression in human neutrophils. J. Leukoc. Biol. 71, 711–717. 10.1189/jlb.71.4.711 11927659

[B132] Nauseef W. M. Borregaard N. (2014). Neutrophils at work. Nat. Immunol. 15, 602–611. 10.1038/ni.2921 24940954

[B133] Netea M. G. Joosten L. A. Latz E. Mills K. H. Natoli G. Stunnenberg H. G.. (2016). Trained immunity: a program of innate immune memory in health and disease. Science 352:aaf1098. 10.1126/science.aaf1098 27102489 PMC5087274

[B134] Nourshargh S. Renshaw S. A. Imhof B. A. (2016). Reverse migration of neutrophils: where, when, how, and why? Trends Immunol. 37, 273–286. 10.1016/j.it.2016.03.006 27055913

[B135] Pandol S. J. Saluja A. K. Imrie C. W. Banks P. A. (2007). Acute pancreatitis: bench to the bedside. Gastroenterology 132, 1127–1151. 10.1053/j.gastro.2007.01.055 17383433

[B136] Paramanathan A. Saxena A. Morris D. L. (2014). A systematic review and meta-analysis on the impact of pre-operative neutrophil lymphocyte ratio on long term outcomes after curative intent resection of solid tumours. Surg. Oncol. 23, 31–39. 10.1016/j.suronc.2013.12.001 24378193

[B137] Pelletier M. Maggi L. Micheletti A. Lazzeri E. Tamassia N. Costantini C.. (2010). Evidence for a cross-talk between human neutrophils and Th17 cells. Blood 115, 335–343. 10.1182/blood-2009-04-216085 19890092

[B138] Peng B. Wang Y. H. Liu Y. M. Ma L. X. (2015). Prognostic significance of the neutrophil to lymphocyte ratio in patients with non-small cell lung cancer: a systemic review and meta-analysis. Int. J. Clin. Exp. Med. 8, 3098–3106. 26064198 PMC4443032

[B139] Peranzoni E. Zilio S. Marigo I. Dolcetti L. Zanovello P. Mandruzzato S.. (2010). Myeloid-derived suppressor cell heterogeneity and subset definition. Curr. Opin. Immunol. 22, 238–244. 10.1016/j.coi.2010.01.021 20171075

[B140] Petit I. Szyper-Kravitz M. Nagler A. Lahav M. Peled A. Habler L.. (2002). G-CSF induces stem cell mobilization by decreasing bone marrow SDF-1 and up-regulating CXCR4. Nat. Immunol. 3, 687–694. 10.1038/ni813 12068293

[B141] Pillay J. Kamp V. M. van Hoffen E. Visser T. Tak T. Lammers J. W.. (2012). A subset of neutrophils in human systemic inflammation inhibits T cell responses through Mac-1. J. Clin. Invest. 122, 327–336. 10.1172/JCI57990 22156198 PMC3248287

[B142] Pillay J. Tak T. Kamp V. M. Koenderman L. (2013). Immune suppression by neutrophils and granulocytic myeloid-derived suppressor cells: similarities and differences. Cell. Mol. Life Sci. 70, 3813–3827. 10.1007/s00018-013-1286-4 23423530 PMC3781313

[B143] Puga I. Cols M. Barra C. M. He B. Cassis L. Gentile M.. (2011). B cell-helper neutrophils stimulate the diversification and production of immunoglobulin in the marginal zone of the spleen. Nat. Immunol. 13, 170–180. 10.1038/ni.2194 22197976 PMC3262910

[B144] Qi H. Yang S. Zhang L. (2017). Neutrophil extracellular traps and endothelial dysfunction in atherosclerosis and thrombosis. Front. Immunol. 8:928. 10.3389/fimmu.2017.00928 28824648 PMC5545592

[B145] Raber P. L. Thevenot P. Sierra R. Wyczechowska D. Halle D. Ramirez M. E.. (2014). Subpopulations of myeloid-derived suppressor cells impair T cell responses through independent nitric oxide-related pathways. Int. J. Cancer 134, 2853–2864. 10.1002/ijc.28622 24259296 PMC3980009

[B146] Raber P. Ochoa A. C. Rodríguez P. C. (2012). Metabolism of L-arginine by myeloid-derived suppressor cells in cancer: mechanisms of T cell suppression and therapeutic perspectives. Immunol. Invest. 41, 614–634. 10.3109/08820139.2012.680634 23017138 PMC3519282

[B147] Ramacciotti E. Hawley A. E. Farris D. M. Ballard N. E. Wrobleski S. K. Myers D. D. Jr.. (2009). Leukocyte- and platelet-derived microparticles correlate with thrombus weight and tissue factor activity in an experimental mouse model of venous thrombosis. Thromb. Haemost. 101, 748–754. 10.1160/TH08-09-0620 19350121 PMC2772897

[B148] Rocha B. C. Marques P. E. Leoratti F. M. S. Junqueira C. Pereira D. B. Antonelli L. R.. (2015). Type I interferon transcriptional signature in neutrophils and low-density granulocytes are associated with tissue damage in malaria. Cell Rep. 13, 2829–2841. 10.1016/j.celrep.2015.11.055 26711347 PMC4698035

[B149] Rodriguez P. C. Quiceno D. G. Zabaleta J. Ortiz B. Zea A. H. Piazuelo M. B.. (2004). Arginase I production in the tumor microenvironment by mature myeloid cells inhibits T-cell receptor expression and antigen-specific T-cell responses. Cancer Res. 64, 5839–5849. 10.1158/0008-5472.CAN-04-0465 15313928

[B150] Saffarzadeh M. Juenemann C. Queisser M. A. Lochnit G. Barreto G. Galuska S. P.. (2012). Neutrophil extracellular traps directly induce epithelial and endothelial cell death: a predominant role of histones. PLoS ONE 7:e32366. 10.1371/journal.pone.0032366 22389696 PMC3289648

[B151] Sagiv J. Y. Michaeli J. Assi S. Mishalian I. Kisos H. Levy L.. (2015). Phenotypic diversity and plasticity in circulating neutrophil subpopulations in cancer. Cell Rep. 10, 562–573. 10.1016/j.celrep.2014.12.039 25620698

[B152] Sagiv J. Y. Voels S. Granot Z. (2016). Isolation and characterization of low- vs. high-density neutrophils in cancer. Methods Mol. Biol. 1458, 179–193. 10.1007/978-1-4939-3801-8_13 27581022

[B153] Scapini P. Cassatella M. A. (2014). Social networking of human neutrophils within the immune system. Blood 124, 710–719. 10.1182/blood-2014-03-453217 24923297

[B154] Schauer C. Janko C. Munoz L. E. Zhao Y. Kienhöfer D. Frey B.. (2014). Aggregated neutrophil extracellular traps limit inflammation by degrading cytokines and chemokines. Nat. Med. 20, 511–517. 10.1038/nm.3547 24784231

[B155] Scheiermann C. Kunisaki Y. Frenette P. S. (2013). Circadian control of the immune system. Nat. Rev. Immunol. 13, 190–198. 10.1038/nri3386 23391992 PMC4090048

[B156] Scheiermann C. Kunisaki Y. Lucas D. Chow A. Jang J. E. Zhang D.. (2012). Adrenergic nerves govern circadian leukocyte recruitment to tissues. Immunity 37, 290–301. 10.1016/j.immuni.2012.05.021 22863835 PMC3428436

[B157] Schmidt H. Bastholt L. Geertsen P. Christensen I. J. Larsen S. Gehl J.. (2005). Elevated neutrophil and monocyte counts in peripheral blood are associated with poor survival in patients with metastatic melanoma: a prognostic model. Br. J. Cancer 93, 273–278. 10.1038/sj.bjc.6602702 16052222 PMC2361564

[B158] Schorn C. Frey B. Lauber K. Janko C. Strysio M. Keppeler H.. (2011). Sodium overload and water influx activate the NALP3 inflammasome. J. Biol. Chem. 286, 35–41. 10.1074/jbc.M110.139048 21051542 PMC3012992

[B159] Schreiber R. D. Old L. J. Smyth M. J. (2011). Cancer immunoediting: integrating immunity's roles in cancer suppression and promotion. Science 331, 1565–1570. 10.1126/science.1203486 21436444

[B160] Semerad C. L. Christopher M. J. Liu F. Short B. Simmons P. J. Winkler I.. (2005). G-CSF potently inhibits osteoblast activity and CXCL12 mRNA expression in the bone marrow. Blood 106, 3020–3027. 10.1182/blood-2004-01-0272 16037394 PMC1895331

[B161] Shah K. Spear J. Nathanson L. A. McCauley J. Edlow J. A. (2007). Does the presence of crystal arthritis rule out septic arthritis? J. Emerg. Med. 32, 23–26. 10.1016/j.jemermed.2006.07.019 17239729

[B162] Silvestre-Roig C. Hidalgo A. Soehnlein O. (2016). Neutrophil heterogeneity: implications for homeostasis and pathogenesis. Blood 127, 2173–2181. 10.1182/blood-2016-01-688887 27002116

[B163] Sionov R. V. Fridlender Z. G. Granot Z. (2015). The multifaceted roles neutrophils play in the tumor microenvironment. Cancer Microenviron. 8, 125–158. 10.1007/s12307-014-0147-5 24895166 PMC4714999

[B164] So A. K. Martinon F. (2017). Inflammation in gout: mechanisms and therapeutic targets. Nat. Rev. Rheumatol. 13, 639–647. 10.1038/nrrheum.2017.155 28959043

[B165] So A. Busso N. (2014). The concept of the inflammasome and its rheumatologic implications. Joint Bone Spine 81, 398–402. 10.1016/j.jbspin.2014.02.009 24703401

[B166] Solito S. Pinton L. Mandruzzato S. (2017). In Brief: myeloid-derived suppressor cells in cancer. J. Pathol. 242, 7–9. 10.1002/path.4876 28097660 PMC5413806

[B167] Spanier B. W. Dijkgraaf M. G. Bruno M. J. (2008). Epidemiology, aetiology and outcome of acute and chronic pancreatitis: an update. Best Pract. Res. Clin. Gastroenterol. 22, 45–63. 10.1016/j.bpg.2007.10.007 18206812

[B168] Ssemaganda A. Kindinger L. Bergin P. Nielsen L. Mpendo J. Ssetaala A.. (2014). Characterization of neutrophil subsets in healthy human pregnancies. PLoS ONE 9:e85696. 10.1371/journal.pone.0085696 24551035 PMC3923728

[B169] Stakos D. A. Kambas K. Konstantinidis T. Mitroulis I. Apostolidou E. Arelaki S.. (2015). Expression of functional tissue factor by neutrophil extracellular traps in culprit artery of acute myocardial infarction. Eur. Heart J. 36, 1405–1414. 10.1093/eurheartj/ehv007 25660055 PMC4458286

[B170] Stark M. A. Huo Y. Burcin T. L. Morris M. A. Olson T. S. Ley K. (2005). Phagocytosis of apoptotic neutrophils regulates granulopoiesis via IL-23 and IL-17. Immunity 22, 285–294. 10.1016/j.immuni.2005.01.011 15780986

[B171] Stephenson H. N. Herzig A. Zychlinsky A. (2016). Beyond the grave: when is cell death critical for immunity to infection? Curr. Opin. Immunol. 38, 59–66. 10.1016/j.coi.2015.11.004 26682763

[B172] Summers C. Rankin S. M. Condliffe A. M. Singh N. Peters A. M. Chilvers E. R. (2010). Neutrophil kinetics in health and disease. Trends Immunol. 31, 318–324. 10.1016/j.it.2010.05.006 20620114 PMC2930213

[B173] Swierczak A. Mouchemore K. A. Hamilton J. A. Anderson R. L. (2015). Neutrophils: important contributors to tumor progression and metastasis. Cancer Metastasis Rev. 34, 735–751. 10.1007/s10555-015-9594-9 26361774

[B174] Talukdar S. Oh D. Y. Bandyopadhyay G. Li D. Xu J. McNelis J.. (2012). Neutrophils mediate insulin resistance in mice fed a high-fat diet through secreted elastase. Nat. Med. 18, 1407–1412. 10.1038/nm.2885 22863787 PMC3491143

[B175] Tecchio C. Cassatella M. A. (2016). Neutrophil-derived chemokines on the road to immunity. Semin. Immunol. 28, 119–128. 10.1016/j.smim.2016.04.003 27151246 PMC7129466

[B176] Tecchio C. Micheletti A. Cassatella M. A. (2014). Neutrophil-derived cytokines: facts beyond expression. Front. Immunol. 5:508. 10.3389/fimmu.2014.00508 25374568 PMC4204637

[B177] Templeton A. J. McNamara M. G. Šeruga B. Vera-Badillo F. E. Aneja P. Ocaña A.. (2014). Prognostic role of neutrophil-to-lymphocyte ratio in solid tumors: a systematic review and meta-analysis. J. Natl. Cancer Inst. 106:dju124. 10.1093/jnci/dju124 24875653

[B178] Terkeltaub R. Baird S. Sears P. Santiago R. Boisvert W. (1998). The murine homolog of the interleukin-8 receptor CXCR-2 is essential for the occurrence of neutrophilic inflammation in the air pouch model of acute urate crystal-induced gouty synovitis. Arthritis Rheum. 41, 900–909. 10.1002/1529-0131(199805)41:5<900::AID-ART18>3.0.CO;2-K 9588743

[B179] Tsuda Y. Takahashi H. Kobayashi M. Hanafusa T. Herndon D. N. Suzuki F. (2004). Three different neutrophil subsets exhibited in mice with different susceptibilities to infection by methicillin-resistant *Staphylococcus aureus*. Immunity 21, 215–226. 10.1016/j.immuni.2004.07.006 15308102

[B180] Ueda Y. Cain D. W. Kuraoka M. Kondo M. Kelsoe G. (2009). IL-1R type I-dependent hemopoietic stem cell proliferation is necessary for inflammatory granulopoiesis and reactive neutrophilia. J. Immunol. 182, 6477–6484. 10.4049/jimmunol.0803961 19414802 PMC2780360

[B181] Uribe-Querol E. Rosales C. (2015). Neutrophils in cancer: two sides of the same coin. J. Immunol. Res. 2015:983698. 10.1155/2015/983698 26819959 PMC4706937

[B182] Van Acker G. J. Saluja A. K. Bhagat L. Singh V. P. Song A. M. Steer M. L. (2002). Cathepsin B inhibition prevents trypsinogen activation and reduces pancreatitis severity. Am. J. Physiol. Gastrointest. Liver Physiol. 283, G794–G800. 10.1152/ajpgi.00363.2001 12181196

[B183] Villanueva E. Yalavarthi S. Berthier C. C. Hodgin J. B. Khandpur R. Lin A. M.. (2011). Netting neutrophils induce endothelial damage, infiltrate tissues, and expose immunostimulatory molecules in systemic lupus erythematosus. J. Immunol. 187, 538–552. 10.4049/jimmunol.1100450 21613614 PMC3119769

[B184] von Vietinghoff S. Ley K. (2008). Homeostatic regulation of blood neutrophil counts. J. Immunol. 181, 5183–5188. 10.4049/jimmunol.181.8.5183 18832668 PMC2745132

[B185] von Vietinghoff S. Ley K. (2009). IL-17A controls IL-17F production and maintains blood neutrophil counts in mice. J. Immunol. 183, 865–873. 10.4049/jimmunol.0804080 19542376 PMC2759196

[B186] Wang J. Sjöberg S. Tang T. T. Oörni K. Wu W. Liu C.. (2014). Cathepsin G activity lowers plasma LDL and reduces atherosclerosis. Biochim. Biophys. Acta 1842, 2174–2183. 10.1016/j.bbadis.2014.07.026 25092171 PMC4188792

[B187] Warnatsch A. Ioannou M. Wang Q. Papayannopoulos V. (2015). Inflammation. Neutrophil extracellular traps licensemacrophages for cytokine production in atherosclerosis. Science 349, 316–320. 10.1126/science.aaa8064 26185250 PMC4854322

[B188] Weaver C. T. Elson C. O. Fouser L. A. Kolls J. K. (2013). The Th17 pathway and inflammatory diseases of the intestines, lungs, and skin. Annu. Rev. Pathol. 8, 477–512. 10.1146/annurev-pathol-011110-130318 23157335 PMC3965671

[B189] Wong S. L. Demers M. Martinod K. Gallant M. Wang Y. Goldfine A. B.. (2015). Diabetes primes neutrophils to undergo NETosis, which impairs wound healing. Nat. Med. 21, 815–819. 10.1038/nm.3887 26076037 PMC4631120

[B190] Woodfin A. Voisin M. B. Beyrau M. Colom B. Caille D. Diapouli F. M.. (2011). The junctional adhesion molecule JAM-C regulates polarized transendothelial migration of neutrophils *in vivo*. Nat. Immunol. 12, 761–769. 10.1038/ni.2062 21706006 PMC3145149

[B191] Wu P. Wu D. Ni C. Ye J. Chen W. Hu G.. (2014). γδT17 cells promote the accumulation and expansion of myeloid-derived suppressor cells in human colorectal cancer. Immunity 40, 785–800. 10.1016/j.immuni.2014.03.013 24816404 PMC4716654

[B192] Xiang M. Yuan Y. Fan L. Li Y. Li A. Yin L.. (2012). Role of macrophages in mobilization of hematopoietic progenitor cells from bone marrow after hemorrhagic shock. Shock 37, 518–523. 10.1097/SHK.0b013e318249b81d 22293600 PMC3328610

[B193] Xu S. Li X. LaPenna K. B. Yokota S. D. Huke S. He P. (2017). New insights into shear stress-induced endothelial signalling and barrier function: cell-free fluid versus blood flow. Cardiovasc. Res. 113, 508–518. 10.1093/cvr/cvx021 28158679 PMC5852521

[B194] Yan J. Kloecker G. Fleming C. Bousamra M. II. Hansen R. Hu X.. (2014). Human polymorphonuclear neutrophils specifically recognize and kill cancerous cells. Oncoimmunology 3:e950163. 10.4161/15384101.2014.950163 25610737 PMC4292216

[B195] Yang F. Feng C. Zhang X. Lu J. Zhao Y. (2017). The diverse biological functions of neutrophils, beyond the defense against Infections. Inflammation 40, 311–323. 10.1007/s10753-016-0458-4 27817110

[B196] Yipp B. G. Petri B. Salina D. Jenne C. N. Scott B. N. Zbytnuik L. D.. (2012). Infection-induced NETosis is a dynamic process involving neutrophil multitasking *in vivo*. Nat. Med. 18, 1386–1393. 10.1038/nm.2847 22922410 PMC4529131

[B197] Youn J. I. Nagaraj S. Collazo M. Gabrilovich D. I. (2008). Subsets of myeloid-derived suppressor cells in tumor-bearing mice. J. Immunol. 181, 5791–5802. 10.4049/jimmunol.181.8.5791 18832739 PMC2575748

[B198] Zenobia C. Hajishengallis G. (2015). Basic biology and role of interleukin-17 in immunity and inflammation. Periodontol. 2000 69, 142–159. 10.1111/prd.12083 26252407 PMC4530463

[B199] Zhang D. Chen G. Manwani D. Mortha A. Xu C. Faith J. J.. (2015). Neutrophil ageing is regulated by the microbiome. Nature 525, 528–532. 10.1038/nature15367 26374999 PMC4712631

[B200] Zitvogel L. Kepp O. Kroemer G. (2010). Decoding cell death signals in inflammation and immunity. Cell 140, 798–804. 10.1016/j.cell.2010.02.015 20303871

